# Fighting Multidrug Resistance with Ruthenium–Cyclopentadienyl Compounds: Unveiling the Mechanism of P-gp Inhibition

**DOI:** 10.1021/acs.jmedchem.3c01120

**Published:** 2023-08-24

**Authors:** Ricardo G. Teixeira, Iris C. Salaroglio, Nuno F. B. Oliveira, João G. N. Sequeira, Xavier Fontrodona, Isabel Romero, Miguel Machuqueiro, Ana Isabel Tomaz, M. Helena Garcia, Chiara Riganti, Andreia Valente

**Affiliations:** † Centro de Química Estrutural, Institute of Molecular Sciences and Departamento de Química e Bioquímica, Faculdade de Ciências, Universidade de Lisboa, Campo Grande, 1749-016 Lisboa, Portugal; ‡ Department of Oncology, University of Torino, 10126 Torino, Italy; § BioISI: Biosystems and Integrative Sciences Institute, Faculdade de Ciências, Universidade de Lisboa, 1749-016 Lisboa, Portugal; ∥ Departament de Química and Serveis Tècnics de Recerca, Universitat de Girona, C/M. Aurèlia Campmany, 69, E-17003 Girona, Spain; ⊥ Molecular Biotechnology Center “Guido Tarone”, University of Torino, 10126 Torino, Italy

## Abstract

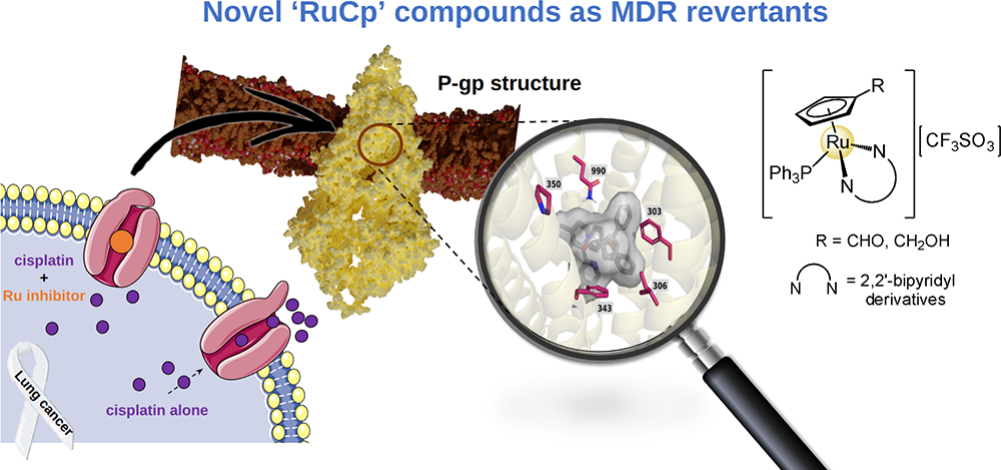

The search for more effective and selective drugs to overcome cancer multidrug resistance is urgent. As such, a new series of ruthenium-cyclopentadienyl (“RuCp”) compounds with the general formula [Ru(η^5^-C_5_H_4_R)(4,4′-R′-2,2′-bipy)(PPh_3_)] were prepared and fully characterized. All compounds were evaluated toward non-small cell lung cancer cells with different degrees of cisplatin sensitivity (A549, NCI-H2228, Calu-3, and NCI-H1975), showing better cytotoxicity than the first-line chemotherapeutic drug cisplatin. Compounds **2** and **3** (R′ = −OCH_3_; R = CHO (**2**) or CH_2_OH (**3**)) further inhibited the activity of P-gp and MRP1 efflux pumps by impairing their catalytic activity. Molecular docking calculations identified the R-site P-gp pocket as the preferred one, which was further validated using site-directed mutagenesis experiments in P-gp. Altogether, our results unveil the first direct evidence of the interaction between P-gp and “RuCp” compounds in the modulation of P-gp activity and establish them as valuable candidates to circumvent cancer MDR.

## Introduction

Multidrug resistance (MDR) mediated by drug efflux pumps is one of the major mechanisms of MDR and severely restricts the long-term use of chemotherapy regimens.^1^ The overexpression of ATP-binding cassette (ABC) transporters in solid tumors has been related to the development of MDR, resulting mostly in an increased drug efflux of first-line chemotherapeutic agents. In fact, the identification of structures that can selectively block such mechanism of efflux is pertinent to be explored and is an attractive strategy to tackle MDR.^2^

**Scheme 1:**
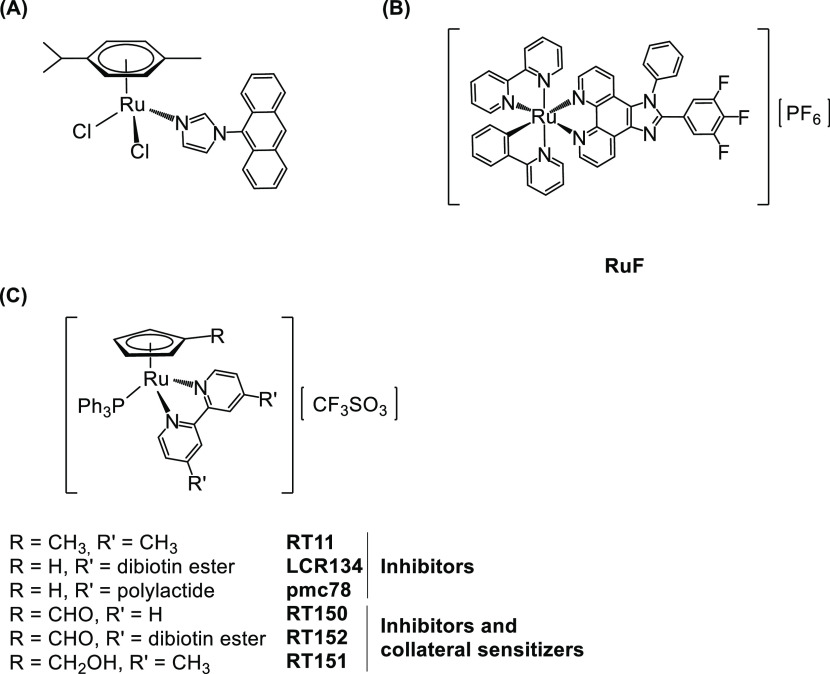
Reported Organometallic Ruthenium ABC Transporter Inhibitors

Over the years, several reports on the evaluation of 1^st^, 2^nd^, and 3^rd^ generation organic-based compounds as inhibitors of the most critical proteins involved in MDR have been reported.^3^ Yet, all of the compounds tested so far failed in preclinical and clinical stages due to their low specificity and high toxicity.^3^ Thus, the search for novel drugs able to treat resistant cancer cells remains a huge challenge. In that frame, the presence of a metal center might be of great benefit for the development of such inhibitors. Juillerat-Jeanneret, Dyson, and co-workers attached modified phenoxazine- and anthracene-based MDR modulator ligands to a ruthenium(II) organometallic scaffold (Scheme 1A).^4^ These compounds were found to be MDR-reverting agents able to inhibit P-gp at 80 μM in the lung cancer A549 cell line, showing similar efficiency as the known P-gp and MRP1 inhibitor verapamil. However, a decrease in *in vitro* selectivity was observed as for many other ABC transporter inhibitors. Another ruthenium compound (RuF, Scheme 1B) was developed by Zeng et al. as a BCRP inhibitor.^5^ In this work, the authors showed that RuF was able to overcome mitoxantrone resistance in lung H460/MX20 cancer cells by downregulating BCRP expression and by inhibiting BCRP ATPase activity. *In vivo* studies in nude mice treated with RuF showed slower tumor growth (*vs* controls) and good tolerability to the drug. Apart from these examples of ruthenium compounds, the copper complex, copper *N*-(2-hydroxy acetophenone)glycinate (CuNG), needs to be mentioned.^6,7^ This compound inhibits drug efflux by direct binding to P-gp. Based on this feature, the same group further developed the manganese and zinc derivatives of CuNG. While ZnNG^8^ was also able to inhibit P-gp, MnNG^9^ was not, thus emphasizing the role of the metal in the inhibition process. Also, a family of cobalt(II)/(III) tris(bipyridine) compounds, in particular [Co(4,4ʼ-dimethyl-2,2ʼ-bipyridine)_3_]^3+^, showed P-gp inhibitory potential.^10^ As a final reference, Domínguez-Álvarez et al. reported a series of selenoanhydrides and selenoesters cytotoxic against MDR mouse T-lymphoma cells^11^ and showing good cancer cell selectivity. Inhibition of P-gp was found to be involved in their mode of action.^12^

During preliminary structure–activity studies for compounds with the general formula [Ru(η^5^-C_5_H_4_R)(PPh_3_)(4,4′-R′-2,2′-bipyridine)]^+^ (Scheme 1C), we observed how subtle changes on the R (substituent at the Cp ring) and R′ (substituent at the bipyridine ligand) drastically changed the performance of the compounds,^13−15^ which can act as P-gp or MRP1 and MRP2 inhibitors or have no inhibitory effect whatsoever. We found out that when R = H and R′ is biotin, the resulting ruthenium compound (**LCR134**) is a strong P-gp inhibitor both *in vitro*(14) and *in vivo*(16) in the zebrafish model. The potency at which the biotinylated compound inhibits P-gp transporters may suggest clinical efficacy at very low doses, potentially reducing off-target effects and increasing compatibility with co-administered chemotherapeutics.

As previously mentioned, one of the major limitations on the development of ABC transporter inhibitors has been the low specificity and high toxicity due to an undesirable accumulation in healthy tissues with central physiological roles disclosed at their preclinical and clinical evaluation.^3^ Alternative approaches have been proposed, such as the design of compounds that are cytotoxic to MDR cells but noncytotoxic to the drug-sensitive parental cells (chemosensitizing agents).^17,18^ This paradoxical hypersensitivity, known as “collateral sensitivity” (CS), creates an “Achilles’ heel”, which can be explored as a target for the development of selective MDR compounds.^19,20^ Some reports have disclosed this potential for a few metal-based compounds,^6,20,21^ yet the molecular basis of this behavior is far from being understood.

We recently disclosed some ruthenium-cyclopentadienyl (“RuCp”) compounds bearing bipyridine-based ligands (Scheme 1C) that showed collateral sensitivity for non-small cell lung cancers (NSCLC).^15^ In this work, we proved that this CS was related to the ability of the compounds to act as ABC pump inhibitors (P-gp and MRP1). Remarkably, when the compounds are administered at nontoxic doses (IC_25_), they can sensitize the resistant cells for treatment with cisplatin (the first-line drug in clinical use) up to 1400-fold. To the best of our knowledge, no other metallodrug has shown such remarkable potency so far. Capitalizing on these results, we decided to further evaluate the role of the substituent at the bipyridine by using a methoxy group. In fact, the methoxy functionality is often found in the chemical structure of several P-gp modulators, and the presence of this hydrogen bond acceptor is probably important for the MDR-reversing activity.^22^ In addition, we aimed to evaluate the presence of biotin at the η^5^-cyclopentadienyl ring *vs* at the bipyridine to conclude this structure–activity study (Scheme S1).

The major goal of this study was to develop more effective “RuCp”-based MDR-reverting agents and explore the mechanism of action of the most promising compounds. *In vitro* cell-based assays and in silico molecular docking calculations were combined to unveil the binding pocket of Ru complexes to P-gp.

## Results and Discussion

### Synthesis and Characterization

**Figure 1:**
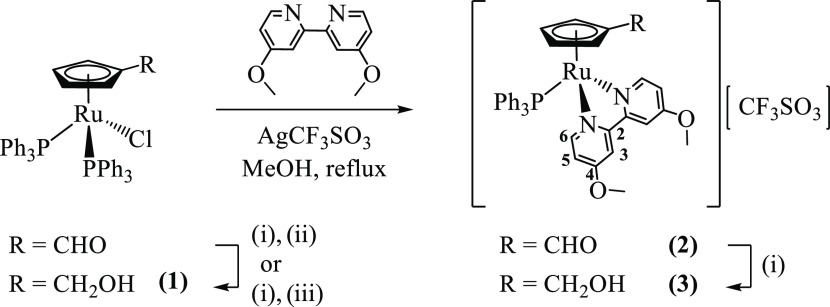
Synthesis of compounds **1**, **2**, and **3**. (i) NaBH_4_, MeOH/tetrahydrofuran (THF); (ii) extraction with CH_2_Cl_2_; and (iii) treatment with chlorinated solvents overnight (e.g., CH_2_Cl_2_ or CHCl_3_).

**Figure 2:**
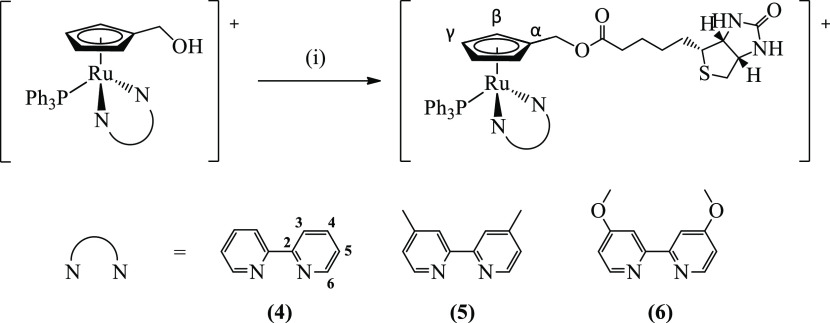
Synthesis of compounds **4**, **5**, and **6**. All compounds were isolated as CF_3_SO_3_^–^ salts. (i) Biotin, EDC·Cl, DMAP, dimethylformamide (DMF), room temperature, overnight. Atom numbering is related to NMR assignments.

We previously reported the synthesis of monofunctionalized ruthenium-cyclopentadienyl compounds of general formula [Ru(η^5^-C_5_H_4_R)(bipy)(PPh_3_)][CF_3_SO_3_] using [Ru(η^5^-C_5_H_4_R)(PPh_3_)_2_X] (R = H, CH_3_, CHO, or CH_2_OH; X = halide) as starting materials.^15,23^ Yet, we found that the compound [Ru(η^5^-C_5_H_4_CH_2_OH)(PPh_3_)_2_Cl] (**1**) can be easily prepared from chlorination of [Ru(η^5^-C_5_H_4_CH_2_OH)(PPh_3_)_2_H] by overnight reaction with CH_2_Cl_2_ or CHCl_3_, or alternatively by direct reduction of [Ru(η^5^-C_5_H_4_CHO)(PPh_3_)_2_Cl] with NaBH_4_, followed by work-up with chlorinated solvents and used as an alternative starting material for the synthesis of ruthenium-cyclopentadienyl compounds with an appended hydroxymethyl group. As such, chloride abstraction with silver trifluoromethanesulfonate from the precursors [Ru(η^5^-C_5_H_4_R)(PPh_3_)_2_Cl] (R = CHO; CH_2_OH (**1**)) followed by sigma coordination of the 4,4′-dimethoxy-2,2′-bipyridine (MeO_2_bipy) ligand in refluxing MeOH led to the formation of compounds **2** and **3**, respectively, in good yields (Figure 1). In addition, an alternative route to prepare compound **3** was also explored by using NaBH_4_ to reduce the formyl substituent of compound **2** at room temperature. The latter proved to be a much more efficient method than the direct coordination of the *N*,*N*-heteroaromatic ligand to [Ru(η^5^-C_5_H_4_CH_2_OH)(PPh_3_)_2_Cl], leading to higher yields and less purification steps (Figure 1). Finally, the addition of biotin on the cyclopentadienyl ring allowed us to isolate three new organometallics structurally related of general formula [Ru(η^5^-C_5_H_4_CH_2_Biotin)(bipy)(PPh_3_)][CF_3_SO_3_] (Figure 2) where bipy is 2,2′-bipyridine (**4**), 4,4′-dimethyl-2,2′-bipyridine (**5**) and 4,4′-dimethoxy-2,2′-bipyridine (**6**) through 4-(*N*,*N*-dimethylamino)pyridine (DMAP)-catalyzed esterification reaction with *N*-(3-dimethylaminopropyl)-*N*′-ethylcarbodiimide hydrochloride (EDC·Cl) in an up to 88% yield. The structure of all new compounds was fully confirmed by NMR (^1^H, ^13^C, and ^31^P nuclei), UV–vis, and Fourier transform infrared (FTIR) spectroscopies, and their purity was assessed by elemental analysis. In addition, the structures proposed for **1**, **2**, and **3** were corroborated by single-crystal X-ray crystallography.

IR spectra in the solid state (KBr pellets) of the new organometallics **1**–**6** showed the presence of the characteristic bands attributed to υ_C–H_ and υ_C=C_ stretching mode of the phosphane, cyclopentadienyl, and (when present) bipyridyl ligands ranging from 3100–3040 and 1490–1390 cm^–1^, respectively. The stretching vibrations of the aldehyde and hydroxy appended groups in compounds **1**, **2**, and **3** were also found at the expected spectral range (1670 cm^–1^ and *c.a.* 3410 cm^–1^, respectively) and the cationic nature of the compounds **2**–**6** was confirmed by the presence of the triflate counterion at the typical region for this group (*c.a.* 1260 cm^–1^). For the biotinylated compounds **4**–**6**, characteristic bands assigned to υ_C=O_ stretching of the ester function (*c.a.* 1710 cm^–1^), as well the υ_N–H_ (3500–3300 cm^–1^) and υ_C–H_ belonging to the alkyl chain of biotin (2930–2860 cm^–1^), were also identified.

All resonances observed in the ^1^H NMR spectra were assigned using uni- and bidimensional experiments and followed the atom numbering presented in Figures 1 and 2. The data collected show resonances for the functionalized η^5^-cyclopentadienyl, bipyridyl, and phosphane in the characteristic range for neutral and monocationic Ru(II) species, respectively (Figures S1–S18). All compounds showed three different resonances, easily ascribed to the three non-equivalent groups of protons of the cyclopentadienyl-derivatized ligand. For compound **1**, two of those resonances appeared at δ = 3.45 and 4.17 ppm and are more deshielded than in the [Ru(η^5^-C_5_H_4_CH_3_)(PPh_3_)_2_Cl] analogue (δ = 3.33 and 3.88 ppm) due to the presence of the primary alcohol moiety at the cyclopentadienyl ring. The hydroxymethyl and −OH protons were observed at δ = 4.35 ppm as a doublet (^3^*J*_HH_ = 6.5 Hz) and δ = 4.49 ppm as a triplet (^3^*J*_HH_ = 6.2 Hz), respectively. Upon coordination of the MeO_2_bipy ligand to both formyl and hydroxymethyl-appended ruthenium-cyclopentadienyl precursors, a deshielding on the H6 protons and a shielding on the H3/4 protons were detected, confirming the coordination of the *N*,*N*-donor to the metal center. Besides, the resonances of the H_β_ and H_γ_ protons of the cyclopentadienyl rings also moved to higher chemical shifts as predicted for the formation of the cationic species. *Ortho* and *meta* couplings for protons H5 and H3 were detected in the value range expected (see the Experimental Section for details). In addition, and in all cases, resonances between 7.45 ppm < δ < 7.11 ppm were observed in all ^1^H NMR complexes’ spectra, which were assigned to the aromatic protons of the triphenylphosphane coligand. The introduction of biotin into the [Ru(η^5^-C_5_H_4_CH_2_OH)(bipy)(PPh_3_)]^+^ fragment *via* esterification was successfully achieved through the “chemistry-on-the-complex” concept and was confirmed by an evident deshielding of the hydroxymethyl protons, as expected by the formation of the corresponding biotin ester. Importantly, this transformation did not affect the coordination of the other coligands and was accompanied by the change in the multiplicity of the -C*H*_2_- protons (that changed from a doublet to a singlet) and the disappearance of −OH proton. Regarding the ^31^P NMR spectra, a sharp singlet resonance corresponding to the coordinated phosphane co-ligand was observed in all cases (δ ∼ 38 ppm for **1**, and δ ∼50–52 ppm for cationic compounds **2**–**6**). The shift observed for the ^31^P NMR (up to ∼14 ppm) upon coordination of the MeO_2_bipy ligand is also in accordance with the formation of cationic structures. A detailed description related to ^13^C NMR experiments for the spectroscopic characterization of the compounds is presented in the Experimental Section, and the results are in agreement with the previously discussed effects in the ^1^H and ^31^P NMR analysis. For all compounds, ^13^C-^31^P couplings were observed in the typical range for aromatic systems and agreed with the expected (2 Hz < ^n^*J*_CP_ < 47 Hz, *n* = 1–4).

**Figure 3:**
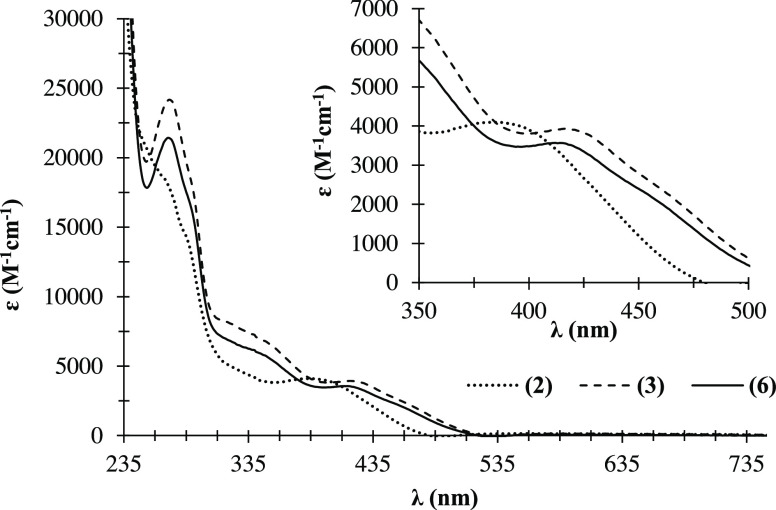
Electronic absorption spectrum in dichloromethane for compounds **2** (**···.**), **3** (- - -), and **6** (^**_____**^) bearing MeO_2_bipy (R = −CHO; −CH_2_OH; −CH_2_Biotin, respectively); inset: MLCT bands.

UV–vis electronic spectra were recorded for all compounds at room temperature using 10^–4^ to 10^–6^ M solutions in dichloromethane and dimethylsulfoxide. Values for the wavelength (λ) and corresponding molar absorptivity coefficient (ε) for the bands observed are collected in Table S1. Figure 3 shows the electronic spectra in dichloromethane solutions of the cationic compounds **2**, **3**, and **6**, which incorporate the MeO_2_bipy ligand and have different substituents at the cyclopentadienyl ligand (R = −CHO; −CH_2_OH; −CH_2_Biotin, respectively). Generally, the spectrum showed two groups of absorption bands: the first one, with two very intense bands in the UV range (λ < 300 nm) assigned to the electronic transitions occurring in the organometallic fragment ({[Ru(η^5^-C_5_H_4_R)(PPh_3_)]^+^}; R = −CHO, −CH_2_OH, −CH_2_Biotin) and the π → π* transitions occurring in the coordinated bipyridyl ligand, respectively; the second (λ 310–360 nm) and third (λ 360 nm up to λ 450 nm) regions may be attributed to the metal-to-ligand charge transfer bands (MLCT). These transitions observed in the latter region are related to transitions from Ru 4d orbitals to π* orbitals of *N*,*N*-heteroaromatic and phosphane coligands, as previously reported for related compounds.^13^ By comparing the spectra of compounds **2**, **3**, and **6**, where the *N*,*N*-heteroaromatic ligand is the same but the cyclopentadienyl substituents change, one can observe that the shift seen in the MLCT band reflects the electronic character of the cyclopentadienyl ligand. Figure 3 shows that complex **2** (bearing the aldehyde substituent) presents the MLCT band occurring at the highest energy, while the MLCT band for compound **3** (with the appended primary alcohol) has the lowest energy; the same trend is observed for related compounds previously reported by us.^15^ In fact, the MLCT band was observed at λ ∼ 390 nm in the spectrum of **2** when compared to **3** and **6** (λ ∼ 420 nm) and related to the stronger donor character of the alcohol/ester compared to the aldehyde substituent at the cyclopentadienyl ring. As expected, the energy of the MLCT band decreased with the increasing donor character of the cyclopentadienyl ring since a better donor would enhance the electronic flow to the Ru(II) cation.^24^

### Crystal Structure of Compounds **1**, **2**, and **3**

**Figure 4:**
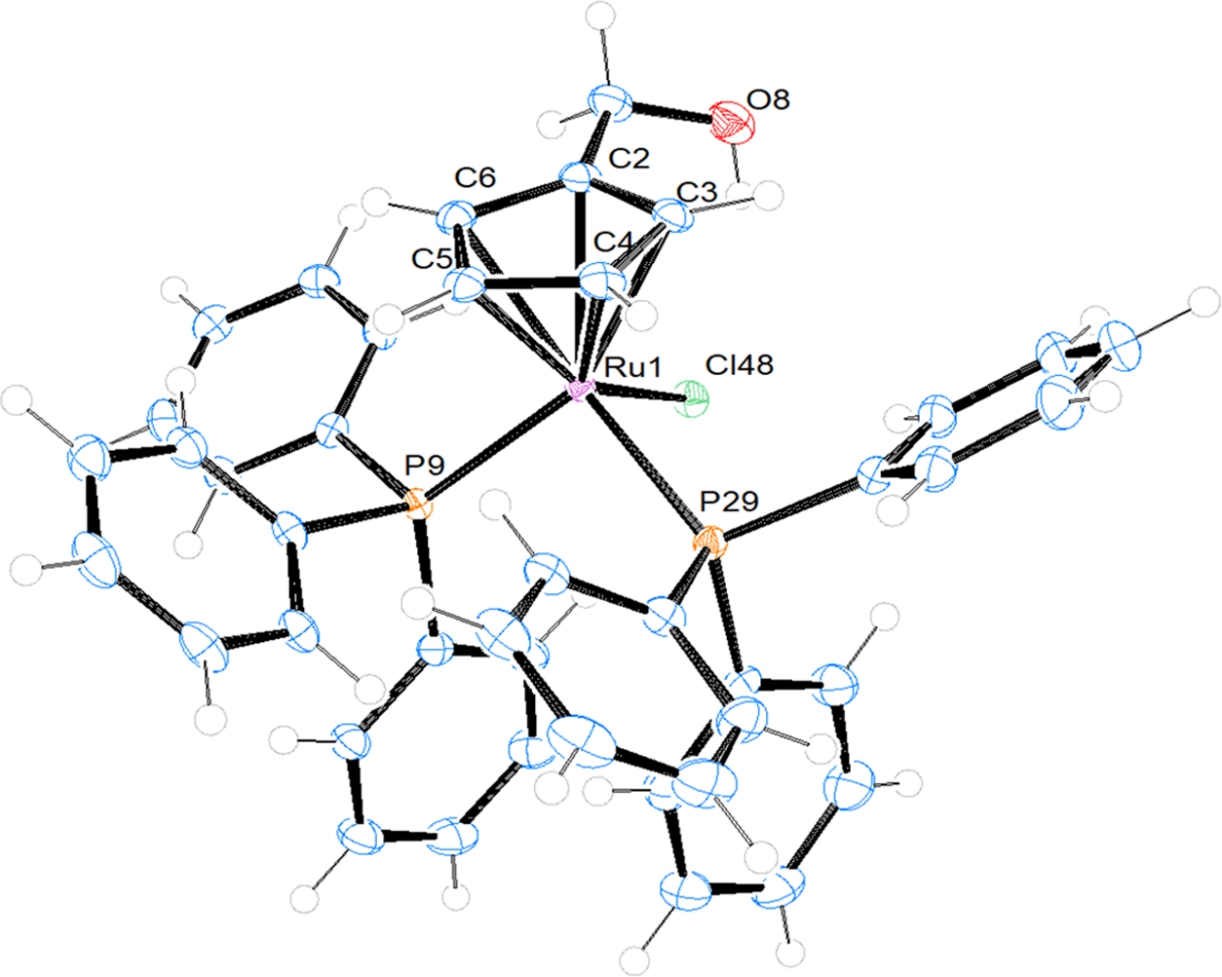
ORTEP plot and labeling scheme of compound **1**. Selected bond lengths (Å), angles (°): Ru1-Cl48, 2.4575(14); Ru1-P9, 2.3220(12); Ru1-P29, 2.3146(13); Ru1-Cp(centroid), 1.849; P9-Ru1-Cl48, 91.92(5); P29-Ru1-Cl48, 90.86(5); P29-Ru1-P9, 99.47(4); Cp(centroid)-Ru1-Cl48, 122.27; Cp-(centroid)-Ru1-P9, 123.14; Cp(centroid)-Ru1-P29, 121.28.

**Figure 5:**
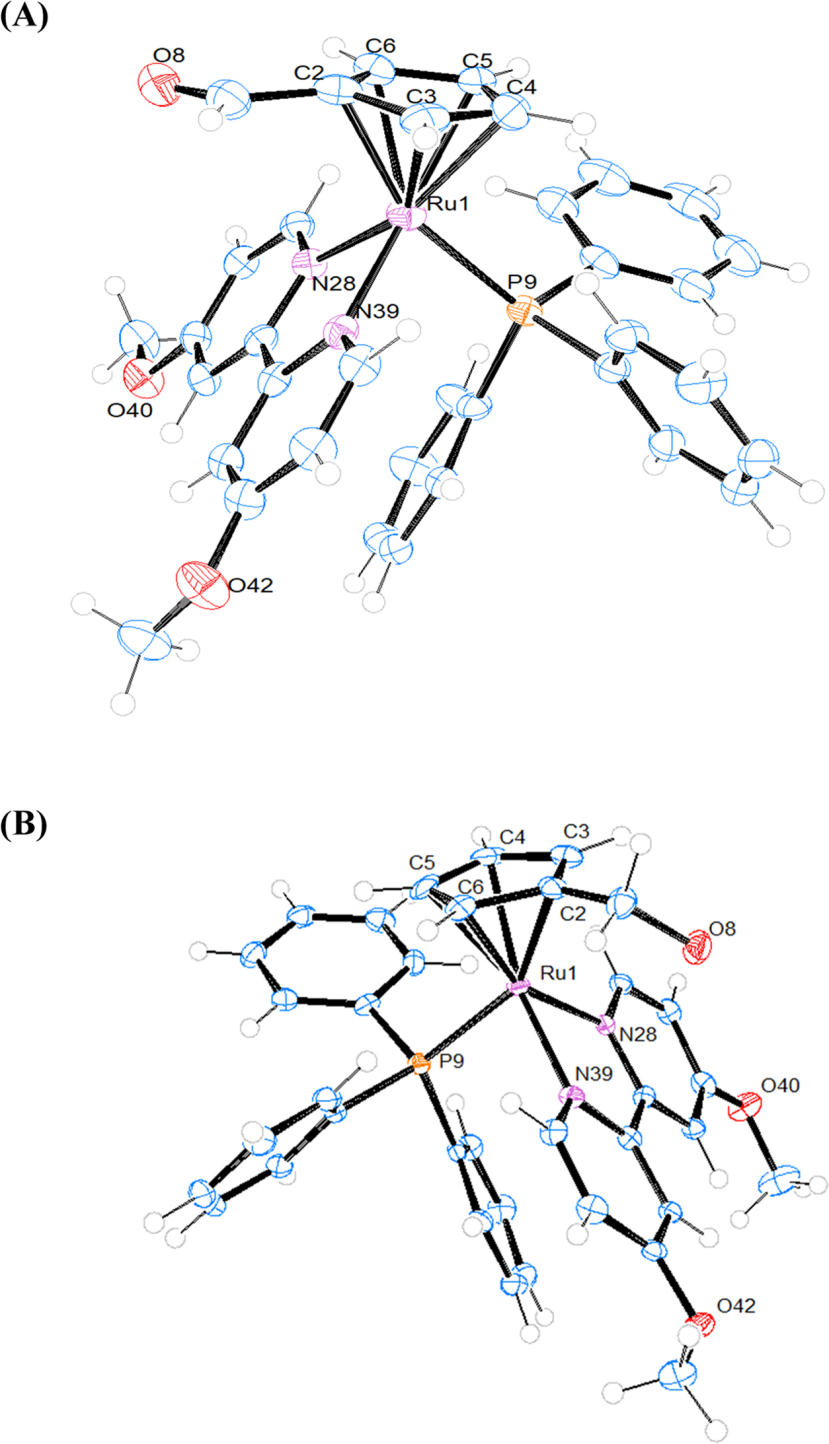
ORTEP plots and labeling schemes for the cations of compounds **2** (A) and **3** (B). Selected bond lengths (Å), angles (°) for **2**: Ru1-P9, 2.3188(10); Ru1-N28, 2.086(3); Ru1-N39, 2.092(3); Ru1-Cp(centroid), 1.820; N28-Ru1-N39, 76.44(12); N28-Ru1-P9, 89.55(9); N39-Ru1-P9, 88.34(9); Cp(centroid)-Ru1-N28, 129.02; Cp-(centroid)-Ru1-N39, 131.21; Cp(centroid)-Ru1-P9, 126.27; for **3**: Ru1-P9, 2.322(2); Ru1-N28, 2.100(3); Ru1-N39, 2.110(3); Ru1-Cp(centroid), 1.832; N28-Ru1-N39, 75.39(12); N28-Ru1-P9, 90.39(8); N39-Ru1-P9, 90.74(8); Cp(centroid)-Ru1-N28, 129.63; Cp-(centroid)-Ru1-N39, 131.77; Cp(centroid)-Ru1-P9, 123.76.

The structures of compounds **1**, **2**, and **3** were confirmed by single-crystal X-ray diffraction analysis. ORTEP views of these structures are shown in Figure 4 (for **1**) and Figure 5 (for **2** and **3**), whereas relevant bond lengths and angles are summarized in the corresponding captions. The main crystallographic data can be found in the Supporting Information (SI) section (Table S2).

Our data confirm the classical piano-stool configuration in which the metal ions are hexacoordinated, surrounded by a monosubstituted η^5^-cyclopentadienyl ring and mono- (**1**) or bidentate ligands (**2** and **3**) occupying the remaining three coordination sites. All compounds crystallize in the triclinic system with the centrosymmetric space group *P*1̅ and the corresponding unit cells of the compounds display two enantiomers in the racemic crystal. In general, the distances Ru–Cp (centroid) are in the same range for all three compounds (1.820–1.849 Å), and the same for the Ru–P (2.3146–2.3222 Å) and Ru–N distances (2.086–2.110 Å) of compounds **2** and **3**. In the case of **1**, the distance Ru–Cl is longer than the Ru–C and Ru–P distances (see captions of Figures 4 and 5). It is worth mentioning the intramolecular hydrogen bonds observed in compound **1**: a strong one between the H atom of the CH_2_OH substituent and the chlorido ligand (H8-Cl48 = 2.362 Å) and two weaker ones between the chlorido ligand and two aromatic hydrogens of each of the two triphenylphosphane ligands (H28-Cl48 = 2.587 Å and H35-Cl48 = 2.643 Å) (see Figure S19). These interactions seem to be responsible for the orientation of the −CH_2_OH substituent on the arene ring. We can also observe that the orientation of substituent CHO on the ring of compound **2** and CH_2_OH in compound **3** is toward the corresponding bipyridine ligands in both cases. Similar behavior has been observed in other cyclopentadienyl complexes described in the literature.^15^ In complex **1**, the P–Ru–P angle is larger than the other two P–Ru–Cl angles, probably due to steric hindrance of the phenyl substituents on both P atoms. The N–Ru–N angles in complexes **2** and **3** show the geometrical restrictions imposed by the bipyridine ligands. The packing structures of the compounds are displayed in Figure S20. Intermolecular hydrogen bonds are observed in **1** between O atoms of the substituents of the η^5^-cyclopentadienyl ligands and the hydrogens of the PPh_3_ ligands of neighboring molecules (O–H = 2.463 Å) (Figure S21A). In the case of **2**, intermolecular hydrogen bonds appear between the F and O atoms of triflate anions and H atoms of the substituents on the bipy and H atoms of the bipy ligands, respectively. Other interactions are observed between O atoms of the arene rings and H atoms of the bpy ligands of neighboring molecules (Figure S21B). In the case of **3**, the triflate anions link two molecules of compound **3** through interactions between their F atoms with H atoms of the arene ring of one molecule and between their O atoms with H atoms of the bipy ligand of the neighboring molecule. Weaker interactions are also observed between O atoms on the substituents of the bipy ligands and hydrogens of the PPh_3_ ligands of neighboring molecules (Figure S21C).

### Stability in Organic and Aqueous Solution

An important feature to address when assessing the biological activity of a new entity is its stability over time because it is essential that the integrity of the molecule is kept until it reaches its intended targets. In this frame, we monitored the stability of all compounds by UV–vis spectroscopy in dimethylsulfoxide (DMSO) and cellular medium (Dulbecco’s modified eagle’s medium (DMEM)). Solutions of compound **1** in DMSO showed to be unstable over time and, for this reason, the biological activity of **1** was not accessed. Complexes **2**–**6** were also tested for their stability in 100% DMSO and in 98% DMEM/2% DMSO. Results indicated that the stability of these structures is adequate for biological evaluation. Figure S22A–E shows the UV–vis spectra along with the variation plot over time for the MLCT band (λ ∼ 380–415 nm) of all compounds measured at specified times during 24 h. In general, the absorption spectra remained roughly the same over time with no significant changes in intensity or in the position and shape of the bands (% variation <10% for all compounds and <∼5% for most, consistent with 90–95% of the parent compound in solution after 24 h), allowing us to pursue the *in vitro* assays in the non-small cell lung cancer model.

### Biological Evaluation of the Compounds

**Figure 6:**
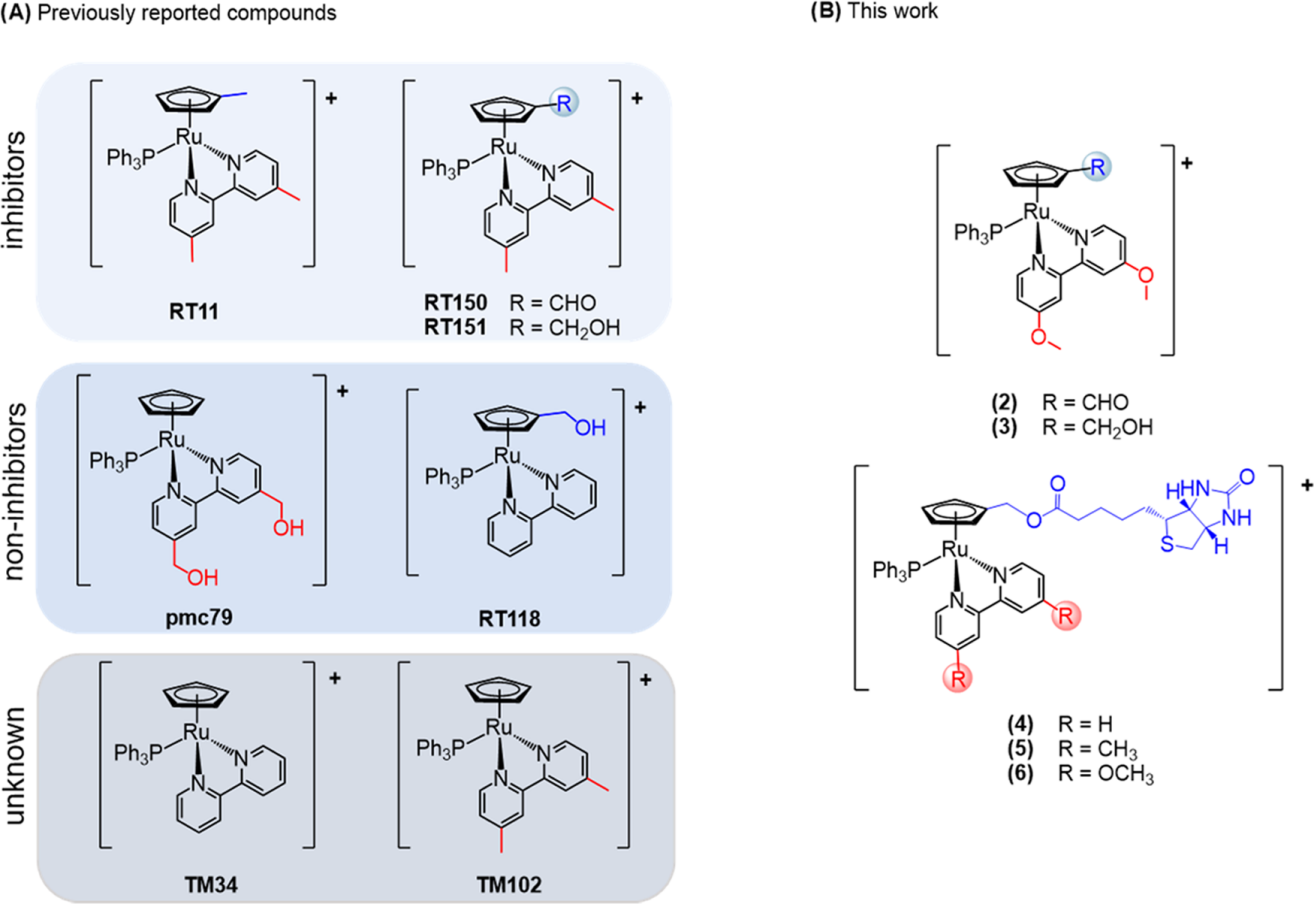
Ruthenium compounds previously described by us (A) and current work (B) used for cell-based assays and molecular docking calculations.

Based on our previous studies on related [Ru(η^5^-C_5_H_4_R)(PPh_3_)(4,4′-R′-2,2′-bipyridine)]^+^ compounds and to further understand the role of substituents at the η^5^-cyclopentadienyl (Cp) ring and at the bipyridine coligands, we enlarged our initial family of compounds to also include: (i) a methoxy function at the bipyridine (compounds **2**–**3**) given the fact that this substituent is frequently present in the structure of compounds with MDR potential;^22^ and (ii) a biotin group at the Cp ring (compounds **4**–**6**) since we previously evidenced the importance of this moiety for P-gp inhibition activity when appended on the bipyridine co-ligand.^14^ In addition, we also determined the IC_50_ for our compounds **RT11**,^13^**TM102**,^25^**RT150**,^15^ and **RT151**(15) previously reported, all bearing the same methyl bipyridine ligand, while changing the substituent at the Cp (Figure 6) to assess the role of the substituent at the Cp on the compound’s activity and possibly on their ability to act as MRP1/P-gp inhibitors. All compounds were incubated at increasing concentrations for 72 h in three non-small cell lung cancer (NSCLC) cell lines resistant to cisplatin (A549, NCI-H2228, and Calu-3 cells) and one cisplatin-sensitive NSCLC cell line (NCI-H1975)^26^ (Table 1). As previously determined by us,^15^ the resistance to cisplatin in these cells is related to the different expression levels of P-gp (Calu-3) and MRP1 (A549 and NCI-H2228) transporters.

**Table 1:** IC_50_ (μM) Values in Lung Cancer Cells Treated with Compounds **2**–**6**, **TM102**, **RT11**, **RT150**, **RT151**, and Cisplatin, after 72 h Incubation with Increasing Concentrations (0–100 μM) of Each Compound, Measured with a Spectrophotometric Assay^a^

compound	A549	NCI-H2228	Calu-3	NCI-H1975
**2**	0.4 ± 0.1	5.6 ± 0.2	3.4 ± 0.9	1.6 ± 0.1
**3**	4.3 ± 0.5	4.1 ± 0.4	7.5 ± 0.8	1.4 ± 0.3
**4**	>100	>100	>100	>100
**5**	>100	>100	>100	>100
**6**	>100	>100	>100	>100
[Ru(η^5^-C_5_H_5_)(Me_2_bipy)(PPh_3_)]^+^ (**TM102**)	7.18 ± 1.28	66.94 ± 7.21	34.19 ± 5.38	44.93 ± 8.75
[Ru(η^5^-C_5_H_4_CH_3_)(Me_2_bipy)(PPh_3_)]^+^ (**RT11**)	32.23 ± 5.56	5.98 ± 0.87	34.17 ± 6.45	49.16 ± 7.12
[Ru(η^5^-C_5_H_4_CHO)(Me_2_bipy)(PPh_3_)]^+^ (**RT150**)	11.3 ± 3.1	3.4 ± 2.1	5.4 ± 1.2	>100
[Ru(η^5^-C_5_H_4_CH_2_OH)(Me_2_bipy)(PPh_3_)]^+^ (**RT151**)	11.6 ± 2.3	9.2 ± 1.6	5.4 ± 1.7	>100
**Cisplatin (CisPt)**	>100	>100	74.9 ± 9.1	4.1 ± 0.8

a Data are means ± standard deviation (SD) (*n* = 3). Compounds with an IC_50_ value of >100 μM are considered inactive (A549, NCI-H2228, and Calu-3: cisplatin-resistant NSCLC lines; NCI-H1975: cisplatin-sensitive NSCLC cell line).

As one can observe, none of the compounds bearing the biotin moiety appended on the Cp ligand was active in any of the lung cancer cells tested, regardless of the substituent at the bipyridine (**4–6**), thus indicating that this functionalization is detrimental for the biological activity of these compounds. This result was somehow surprising given the fact that our previous studies identified the compound [Ru(η^5^-Cp)(PPh_3_)(2,2′-bipy-4,4′-dibiotin ester)]^+^ (**LCR134**) as cytotoxic for several cancer cell lines and as a P-gp inhibitor.^14,27^ In addition, previous molecular dynamics (MD) simulations indicated that derivatizations on either 2,2′-bipy or Cp should not perturb the membrane interaction modes of [Ru(η^5^-C_5_H_4_R)(PPh_3_)(4,4′-R′-2,2′-bipyridine)]^+^ structures since those, unlike PPh_3_, were the ones more accessible to the water phase. Nevertheless, there is the possibility that the biotin derivatization specifically on Cp is not altering the biological membrane interaction profile but leads to a complex that has a size/shape and polarity inadequate to bind its preferred protein target.

It is interesting to observe that while compounds **RT150** and **RT151** (R′ = methyl) previously reported were selective for cisplatin-resistant cells, compounds **2** and **3**, bearing a methoxy substituent at the bipyridine instead of methyl, are active against all cell lines tested, including those sensitive to the treatment with cisplatin. As such, they are not inducers of collateral sensitivity, and their mechanisms of action should be different from that of **RT150** and **RT151**.

Comparing all of the compounds bearing methyl bipyridine but with different substituents at the Cp ligand, we can conclude that the functionalization with the formyl (**RT150**) or hydroxyl (**RT151**) groups somehow leads to a selectivity toward the resistant cells, while those with methyl (**RT11**) or not at all substituted (**TM102**) lead to compounds that are only moderately cytotoxic in all cancer cell lines tested.

Overall, these results highlight the contribution of the formyl and hydroxyl functionalization at the Cp for the selectivity and activity of the compounds, for which activity is also positively tuned by the substituent at the bipyridine co-ligand (R′): the most active compounds exhibit the methoxy functionality (**2** and **3**) and compounds bearing the methyl group (**RT150** and **RT151**) are inducers of collateral sensitivity.

Next, we focused on the most cytotoxic compounds **2** and **3**, and we evaluated if they were able to sensitize resistant cell lines to cisplatin. As expected, the IC_50_ of cisplatin in intrinsically chemoresistant A549, NCI-H2228, and Calu-3 cells was higher than the IC_50_ in NCI-H1975 (Table 2). Both compounds **2** and **3** dramatically decreased the IC_50_ of cisplatin in the resistant cell lines when administered at a nontoxic dose (0.1 μM), while they have little-to-no effect on cisplatin cytotoxicity on (drug-sensitive) NCI-1975 cells.

**Table 2:** IC_50_ (μM) of Cells Measured after 72 h Incubation with Increasing Concentrations (0–100 μM) of Cisplatin (CisPt), Alone or Coincubated with 0.1 μM of Compounds **2**, **3**, **RT150** and **RT151**, Measured with a Spectrophotometric Assay^a^^,^^b^

compounds	A549	NCI-H2228	Calu-3	NCI-H1975
**CisPt**	>100	29.87 ± 4.87	>100	1.86 ± 0.3
**CisPt+2**	2.9 ± 0.5***	2.2 ± 0.3***	0.16 ± 0.05***	1.36 ± 0.5
**CisPt+3**	2.0 ± 0.3***	1.4 ± 0.5***	0.63 ± 0.08***	2.66 ± 0.3
**CisPt**+RT150	4.2 ± 0.4***	3.2 ± 0.4***	1.03 ± 0.13***	2.01 ± 0.3
**CisPt**+RT151	3.6 ± 0.6***	2.8 ± 0.6***	1.21 ± 0.17***	1.87 ± 0.5

a Data are means ± SD (*n* = 3).

b ***:*p* < 0.001: vs PT alone.

This great sensitizer potential found for **2** and **3** was similar to the effect exerted by **RT150** and **RT151**, which we already showed to inhibit the MRP1 ATPase catalytic cycle and ATP-driven catalytic efflux in A549 and NCI-H2228 cells and also on P-gp activity in Calu-3 cells.^15^

**Figure 7:**
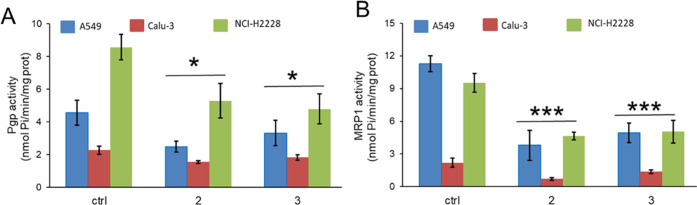
P-gp (A) and MRP1(B) ATPase activity, measured spectrophotometrically on the proteins immune-purified from A549, NCI-H2228, and Calu-3 cells, respectively, treated without (ctrl) or with 0.1 μM of compounds **2** or **3** for 3 h. Data are means ± SD (*n* = 3). *p < 0.05, ***p < 0.001: *vs* ctrl.

The main reason for chemoresistance to cisplatin in the three cell lines analyzed is the presence of ABC transporters, as previously mentioned.^26^ Notably, compounds **2** and **3** inhibited the activity of P-gp and MRP1 (Figure 7A,B), with a potency superimposable to that of **RT150** and **RT151** in the same cell lines.^15^

Overall, these results are clear evidence of the importance that the ABC transporters surely have on the mechanism of action for these compounds, in particular for **RT150** and **RT151**, which are only active against cells overexpressing MRP1 and P-gp. As such, to better understand the molecular details of a possible interaction between the compounds and the ABC transporters, we chose P-gp as a model for molecular docking calculations. The use of MRP1 in our computational studies was unfeasible due to the absence of a reliable experimental structure.

#### Molecular Docking Calculations to Estimate P-gp Binding Affinities of the Ru Complexes and Experimental Verification

**Figure 8:**
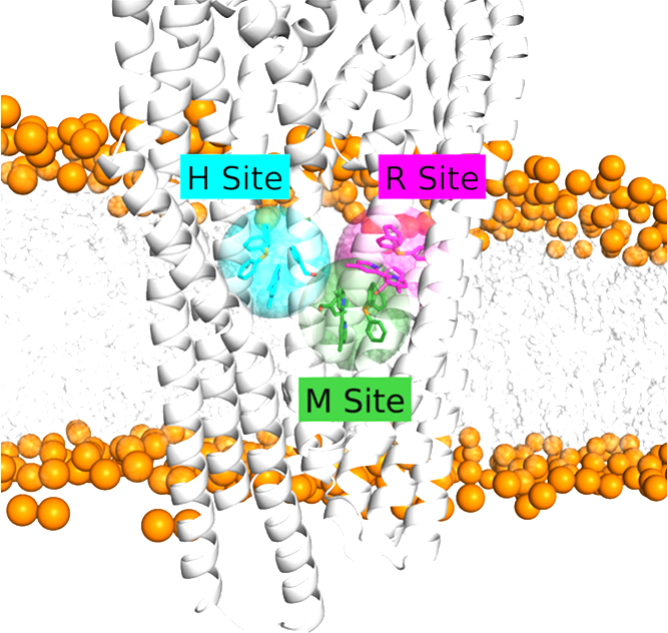
P-gp structure representation with R, M, and H binding sites highlighted (magenta, green, and cyan, respectively) with an example of the bound Ru complex. P-gp was obtained from AlphaFold database (P08183)^28^ and is shown as a gray cartoon inserted in a POPC membrane with the phosphate groups shown as orange spheres and acyl chains as gray sticks.

We performed molecular docking calculations using several ruthenium-based complexes, which are known P-gp inhibitors,^14,15^ non-inhibitors,^13−15^ or unknown. The calculations were done separately on the 3 reported binding sites of P-gp: R, M, and H (Figure 8), and the binding energies for the best binding poses are reported in Table 3. The results show that all compounds prefer the R-site, which is located at the membrane/P-gp interface and can be seen as the exporter entry point for membrane-inserted compounds. On the other hand, the H-site is unfavored for most compounds, which may be related to its smaller size and the presence of slightly more polar residues. Notwithstanding, the lack of flexibility in the residue side chains in our docking protocol can also be influencing these preferences.

**Table 3:** Molecular Docking Binding Energies of all Ru Complexes in Different P-gp Binding Sites^a^

		binding energy		
Ru complex		R-site	M-site	H-site
inhibitor	[Ru(η^5^-C_5_H_4_CHO)(Me_2_bipy)(PPh_3_)]^+^**RT150**	–9.5	–8.3	–7.9
	[Ru(η^5^-C_5_H_4_CH_2_OH)(Me_2_bipy)(PPh_3_)]^+^**RT151**	–9.3	–7.9	–8.2
	[Ru(η^5^-C_5_H_4_CHO)(MeO_2_bipy)(PPh_3_)]^+^**2**	–9.0	–8.2	–7.4
	[Ru(η^5^-C_5_H_4_CH_2_OH)(MeO_2_bipy)(PPh_3_)]^+^**3**	–8.8	–7.6	–7.3
non-inhibitor	[Ru(η^5^-C_5_H_4_CH_2_OH)(bipy)(PPh_3_)]^+^**RT118**	–8.7	–8.1	–7.2
	[Ru(η^5^-C_5_H_5_)(bipy(CH_2_OH)_2_)(PPh_3_)]^+^**PMC79**	–8.6	–7.6	–7.4
unknown	[Ru(η^5^-C_5_H_5_)(bipy)(PPh_3_)]^+^**TM34**	–8.8	–8.2	–8.5
	[Ru(η^5^-C_5_H_5_)(Me_2_bipy)(PPh_3_)]^+^**TM102**	–9.4	–9.1	–7.6

a The inhibitors, non-inhibitors, and complexes with unknown activity are grouped. All binding energies are shown in kcal/mol.

Although the molecular docking results could not categorically distinguish between inhibitors and non-inhibitors, probably due to limitations in the Autodock Vina scoring functions dealing with these very similar structures, they still provided the preferred binding poses for each compound, which can help us identify the key P-gp residues in the ruthenium complex binding.

From all of the conformations obtained in the docking protocol, an analysis of P-gp residues located ∼5 Å from our ligand was performed to identify proximal contacts (Table S3 and Figure S23 of the SI). Unsurprisingly, the residues located around the binding modes across all binding pockets have a hydrophobic character (Phe, Trp, Ile, Leu, and Tyr). Some of these residues will form stabilizing π-stack and/or hydrophobic interactions with the Ru complexes and can have an important role in the inhibition mechanism of P-gp.

Interestingly, some complexes displaying aldehyde and hydroxyl substitution on the Cp group of the Ru complex seem to establish stable interactions with glutamine residues (Figure S23 of the SI). To evaluate the individual role of each of these residues, we sequentially mutated them to alanine. The resulting structures were then docked with **RT151** (inhibitor) and **RT118** (non-inhibitor), and the calculated binding energies were compared to the ones obtained in the wild-type P-gp (Tables S4 and S5 of the SI).

From this protocol, we are interested in identifying residues that have a significant impact on the preferred binding mode independently of the stabilization/destabilization effect. The main goal is to select P-gp residues that, when mutated experimentally, can lead to a change in the effect of the Ru complex on the activity of the protein and, consequently, assign unequivocally the correct binding pocket for the tested compound. To carefully select these residues, we must consider the nature of the residue and if the difference in the binding energy came from a loss/gain of interaction or simply due to conformational restraints. The latter should not have a large influence on the decision due to the lack of structural flexibility in our docking protocol. Nevertheless, the most promising residue positions were identified and tested experimentally in activity assays using the respective P-gp mutants (Table 4). We started by measuring the ATPase activity of P-gp in MDCK-P-gp-overexpressing cells, which are devoid of MRP1, to maximize the potential interactions between the compounds and P-gp. As shown in Table 4, compounds **2** and **3** exerted similar P-gp inhibition activities as **RT150** and **RT151**, while—as expected—the substrate **RT118** had no effects.

**Table 4:**
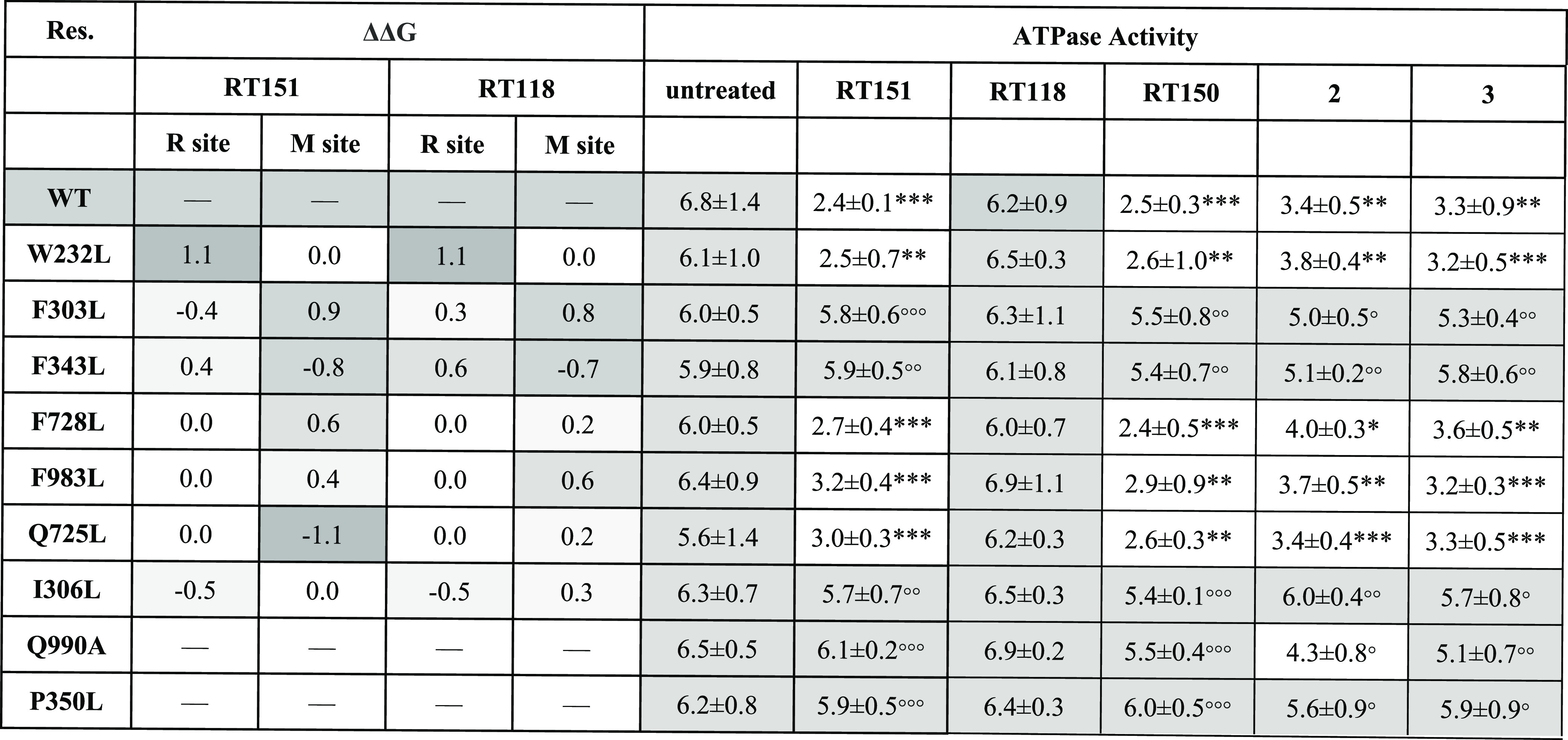
Changes in Binding Energies (ΔΔ*G*) of the Most Promising P-gp Mutations that were Selected to be Tested Experimentally^a^^,^^b^

a The binding energy deviations (in kcal/mol) caused by the Ala mutation of each residue are color-coded according to their magnitude as a visual aid. The ATPase activity of P-gp extracted from MDCK-P-gp cells, bearing wild-type or mutated P-gp, treated for 3 h with 0.1 μM of the indicated compounds is reported in nmol (inorganic phosphate)·min^–1^·mg^–1^(protein). Data are means ± SD (*n* = 3).

b WT: wild-type; *p < 0.05, **p < 0.01, ***p < 0.001: vs untreated cells expressing WT P-gp; °*p* < 0.05, °°*p* < 0.01, °°°*p* < 0.001: vs cells expressing WT P-gp, treated with the corresponding compound.

The P-gp residues that were mutated are part of the R-site, the M-site, or both (located at the interface). In our first experiments, we identified W232, F303, F343, F728, F983, Q725, and I306 as key residues in the binding affinity of the inhibitor **RT151**. Since the best binding modes between all our compounds from molecular docking calculations are relatively similar, it results that the same residues also influence the binding of the non-inhibitor **RT118**. Therefore, we propose that the differences between inhibitors and non-inhibitors are subtle structural changes that provide enhanced binding affinities and result in more stable P-gp/Ru-compound complexes, hindering the normal efflux function of the protein. The ATPase activity experiments in the presence of **RT151** confirmed that only residues located in the R-site of P-gp (F303, F343, and I306) destabilized the P-gp/**RT151** complex to recover the activity values of the wt untreated protein (6.8 ± 1.4 nmol·min^–1^·mg^–1^). The W232 residue from the R-site was identified in the ala-scanning protocol; however, it did not impact the P-gp activity, indicating that the real binding mode should not depend on this residue. We extended the ATPase activity experiments to the other known P-gp inhibitors (**RT150**, **2**, and **3**) and to the non-inhibitor (**RT118**), which confirmed a similar pattern for all inhibitors and a lack of effect for **RT118** (Table 4).

**Figure 9:**
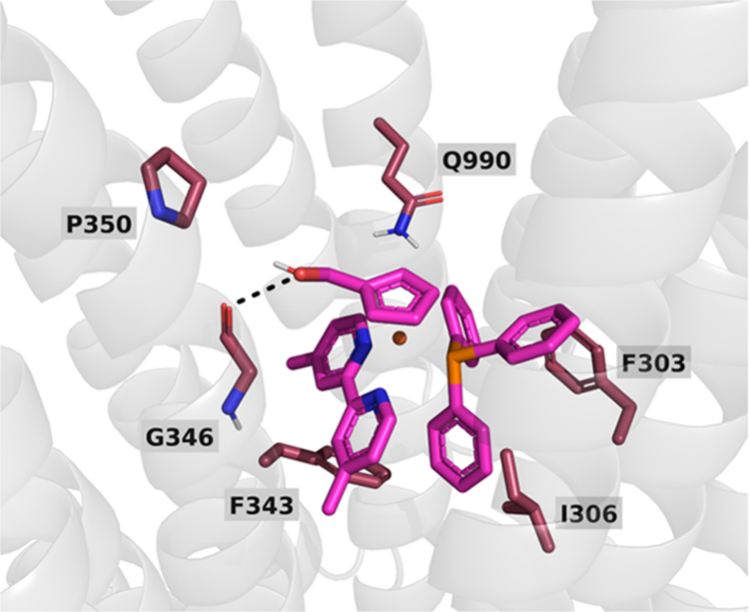
Key P-gp residues in the R-site that are involved directly or indirectly in the stabilization of the Ru-complex binding. The 6 residues (G346, P350, F303, I306, F343, and Q990) are shown in raspberry color sticks, and the **RT151** is represented in magenta sticks. The key H-bond interaction with the G346 carbonyl group is marked as a black dashed line.

From the information obtained in the first experimental setting, we went back to the original binding poses of **RT151** to identify poses that were stabilized by residues F303, F343, and I306 and without a critical role of W232. In the most promising configurations, **RT151** was also found to establish interactions with the side chain of residue Q990 and the main-chain carbonyl group of residue G346 (Figure 9). This glycine residue is located in the middle of a transmembrane helix, and its main-chain carbonyl group is available to interact with ligands because the residue located in the +4 position of the α-helix is a proline (P350), which does not establish the canonical hydrogen bond and generates a gap/kink in the transmembrane helix (Figure 9). In our docking pose, we observed a clear interaction of the **RT151** hydroxyl group with the G346 carbonyl group and devised a strategy to confirm the role of this residue. We proposed the mutation of P350 to induce the formation of the 346–350 hydrogen bond and block the interaction with the ligand. The experimental data on P-gp activity confirmed our binding pose since the mutations on residues Q990 and P350 led to an almost complete recovery of the protein activity (Table 4).

In summary, we have identified the preferred binding pocket in P-gp for all Ru-complex derivatives that exhibit an inhibitory effect on the exporter. Additionally, we used site-directed mutagenesis to validate our molecular docking data and help identify the most promising binding poses. This information will be a very important aid for the future rational design of new Ru-complex derivatives, tailor-made to maximize the affinity for the R-site of P-gp.

## Conclusions

The preparation and characterization of [Ru(η^5^-C_5_H_4_CH_2_OH)(PPh_3_)_2_Cl] (**1**) and two structurally related sets of compounds of general formula [Ru(η^5^-C_5_H_4_R)(MeO_2_bipy)(PPh_3_)]^+^ (where R = CHO (**2**), CH_2_OH (**3**)) and [Ru(η^5^-C_5_H_4_CH_2_Biotin)(bipy)(PPh_3_)]^+^ (with bipy = 2,2′-bipyridine (**4**), 4,4′-dimethyl-2,2′-bipyridine (**5**) and 4,4′-dimethoxy-2,2′-bipyridine (**6**)) was successfully performed by several analytical and spectroscopic methods. The X-ray structures of compounds **1**–**3** display hexacoordinated Ru(II) ions in full agreement with the structures shown in solution. All cationic compounds, together with **RT150**, **RT151**, **RT11**, and **TM102** (that we previously reported), were studied as potential anticancer agents for the treatment of non-small lung cancer in a panel of cells with different resistance extents to the treatment with cisplatin. Biotin-based compounds were inactive against all cell lines tested, while the new compounds **2** and **3** were highly active against all NSLC cells. In addition, and similarly to compounds **RT150** and **RT151**, **2**, and **3** were remarkably competent at sensitizing all resistant cells toward the treatment with cisplatin when administered at nontoxic doses. Therefore, compounds **2** and **3** emerged as new chemosensitizing agents in NSCLC cells. The molecular mode of interaction with P-gp was also assessed. Mutagenesis experiments coupled with molecular docking calculations allowed the identification of the P-gp binding pocket for these Ru complexes. To the best of our knowledge, this is the first report containing such high molecular detailed information about the development of metallodrugs as anticancer agents. This will serve as an essential instrument to direct future synthesis toward the fine-tuning of new ruthenium-cyclopentadienyl P-gp inhibitors. Among all of the tested compounds, **RT150** and **RT151** emerge as lead compounds due to their ability to act as collateral sensitizers, i.e., they are selectively cytotoxic to MDR cells but noncytotoxic to the drug-sensitive parental cells.

Results reported herein, together with our previous work on Ru–Cp complexes in the context of drug resistance, definitely establish these compounds as candidates of exceptional value to circumvent MDR in cancer therapy, encompassing a dual action as cytotoxic metallodrugs highly active against lung cancer as well as competent chemosensitizing agents in resistant NSCLC cells when used at a lower noncytotoxic dose. The great translational potential that these features confer to these lead compounds cannot be overstated, given the extent of chemoresistance to the first-line cisplatin treatment that occurs in up to 60% of cancer treatment regimens currently available.

## Experimental Section

### Materials

Unless otherwise stated, all chemicals were purchased from commercial sources and used without further purification. The ruthenium compounds of general formula [Ru(η^5^-C_5_H_4_CHO)(PPh_3_)_2_(L)] (where L = Cl; H),^23^ [Ru(η^5^-C_5_H_4_R)(Me_2_bipy)(PPh_3_)][CF_3_SO_3_] (with R = H, **TM102**;^25^ R = CH_3_, **RT11**;^13^ R = CHO, **RT150**;^15^ R = CH_2_OH, **RT151**(15) and Me_2_bipy is 4,4′-dimethyl-2,2′-bipyridine) were prepared by using the methods previously described by us.

### Instrumentation and Methods (Experimental Section)

#### General Procedures

All reactions and purification of compounds were performed under a nitrogen atmosphere using Schlenk techniques. All solvents were used as purchased, with the exception of dichloromethane, *n*-hexane, and tetrahydrofuran used for synthetic procedures and work-up, which were dried using an MBRAUN solvent purification system (MB SPS-800, M Braun Inertgas-Systeme GmbH, Garching, Germany). NMR spectra were recorded on a Bruker Avance 400 spectrometer at probe temperature using commercially available deuterated acetone. Chemical shifts (δ) are reported in parts per million (ppm) referenced to tetramethylsilane (δ 0.00 ppm) using the residual proton solvent peaks as internal standards. The multiplicity of the peaks is abbreviated as follows: s (singlet), d (doublet), t (triplet), m (multiplet), and comp (complex). Coupling constants (*J*) are reported in Hertz (Hz). All assignments were attributed using COSY, HMBC, and HMQC 2D-NMR techniques. Infrared spectra were recorded on KBr pellets using a Mattson Satellite FTIR spectrophotometer. Only bands considered relevant were cited in the text. Electronic spectra were recorded at room temperature on a Jasco V-660 spectrometer from solutions of 10^–4^–10^–6^ M in quartz cuvettes (1 cm optical path). The purity of all complexes was assessed by high-performance liquid chromatography (HPLC) and elemental analysis. All compounds are >95% pure by HPLC (see the SI). HPLC analysis was performed on an Ultimate 3000 Dionex system (Dionex Co., Sunnyvale, CA) using a Luna C18 (2) column (250 mm × 4.6 mm; 5 μm; Phenomenex, Torrance, CA). Elemental analyses were performed at *Laboratório de Análises*, at *Instituto Superior Técnico*, using a Fisons Instruments EA1 108 system.

### Synthesis of the Ruthenium Complexes

#### [Ru(η^5^-C_5_H_4_CH_2_OH)(PPh_3_)_2_Cl] (**1**)

Method A: To a mixture of [Ru(η^5^-C_5_H_4_CHO)(PPh_3_)_2_Cl] (250 mg, 0.3 mmol) and NaBH_4_ (560 mg, 14.8 mmol) in THF (2 mL), MeOH (5 mL) was slowly added over 30 min. Following the addition, the reaction mixture was further stirred at room temperature until gas bubbles were no longer detected (approximately 60 min). The solvents were removed under vacuum, and the residue obtained was extracted with dichloromethane (20 mL × 3) and chloroform (10 mL × 2) and filtered through Celite. After extraction, the filtrate was concentrated to dryness, washed with hexane (5 mL × 3), and then recrystallized from slow diffusion of *n*-hexane in a solution of toluene to afford a dark orange crystalline solid. Yield: 42% (105 mg).

Method B: Alternatively, compound **1** can be obtained from the chlorination reaction of [Ru(η^5^-C_5_H_4_CH_2_OH)(PPh_3_)_2_H] with CH_2_Cl_2_ or CHCl_3_, at room temperature and in almost quantitative yield (*c*.*a*. 95%).

^1^H NMR [(CD_3_)_2_CO, Me_4_Si, δ/ppm]: 7.40 (m, 12H, H_*ortho*_-PPh_3_), 7.31 (m, 6H, ^3^*J*_HH_ = 7, H_*para*_-PPh_3_), 7.20 (m, 12H, ^3^*J*_HH_ = 7.4, H_*meta*_-PPh_3_), 4.49 (t, 1H, ^3^*J*_HH_ = 6.2, C_5_H_4_CH_2_OH), 4.35 (d, 2H, ^3^*J*_HH_ = 6.4, C_5_H_4_CH_2_OH), 4.17 (broad, 2H, H_β_-C_5_H_4_CH_2_OH), 3.45 (s, 2H, H_γ_-C_5_H_4_CH_2_OH). APT-^13^C{^1^H} NMR [(CD_3_)_2_CO, δ/ppm]: 139.4 (d, ^1^*J*_PC_ = 39.3, C_q_-PPh_3_), 134.7 (t, ^2^*J*_PC_ = 5, CH_*ortho*_-PPh_3_), 129.8 (CH_*para*_*-*PPh_3_), 128.3 (t, ^3^*J*_PC_ = 4.5, CH_*meta*_-PPh_3_), 78.1 (t, ^2^*J*_PC_ = 4.5, C_β_-C_5_H_4_CH_2_OH), 76.9 (C_γ_-C_5_H_4_CH_2_OH), 59.5 (C_5_H_4_CH_2_OH). ^31^P{^1^H} NMR [(CD_3_)_2_CO, δ/ppm]: 38.4 (s, PPh_3_). FTIR [KBr, cm^–1^]: 3412 (υ_O-H_), 2848 (υ_C-H_ alkanes), 3075–3057 (υ_C-H_ Cp and aromatic rings), 2848 cm^–1^ (υ_C-H_ alkanes), 1480 (υ_C=C_ aromatic). UV–vis [CH_2_Cl_2_, λ_max_/nm (ε × 10^3^/M^–1^ cm^–1^)]: 290 (sh), 355 (sh), 373 (2390), 450 (sh). Elemental analysis calcd for C_42_H_37Cl_OP_2_Ru (756.21): C, 66.71 H, 4.93. Found: C, 66.8; H, 5.0.

#### [Ru(η^5^-C_5_H_4_R)(4,4′-CH_3_O-2,2′-bipy)(PPh_3_)][CF_3_SO_3_] (**2** and **3**)

Method A: Treatment of [Ru(η^5^-C_5_H_4_R)(PPh_3_)_2_Cl] (R = CHO 113 mg; R = CH_2_OH 113 mg; 0.15 mmol) with silver trifluoromethanesulfonate (51 mg, 0.20 mmol) in degassed methanol (15 mL) and in the presence of a slight excess (1.2 equiv) of 4,4′-dimethoxy-2,2′-bipyridine (40 mg, 0.18 mmol), at reflux under a nitrogen atmosphere for 14 h. After cooling to room temperature, filtering, and removing the solvent, the crude solid was treated with a mixture of propan-2-ol/water (v/v 1: 2, 15 mL) and filtered. The filtrate was concentrated to dryness, and the residue obtained was washed with *n*-hexane (15 mL × 3) and recrystallized from acetone/*n*-hexane to give dark orange crystals.

Method B: Alternatively, compound **3** can be obtained in higher yields starting from **2**. To a mixture of **2** (0.100 mg, 0.12 mmol) and NaBH_4_ (380 mg, 10 mmol) in THF (2 mL), MeOH (8 mL) was slowly added over 30 min. Following the addition, the mixture was stirred at room temperature until gas bubbles were no longer detected (*ca.* 90 min). After that, volatiles were removed under vacuum, and the residue obtained was extracted with dichloromethane (10 mL × 4) and filtered through Celite. The filtrate was concentrated to dryness, and the residue was washed with water (5 mL × 3) and *n*-hexane (5 mL × 3) and then recrystallized by slow diffusion of *n*-hexane into a dichloromethane solution of **3** to afford dark orange crystals.

#### [Ru(η^5^-C_5_H_4_CHO)(4,4′-OCH_3_–2,2′-bipy)(PPh_3_)][CF_3_SO_3_] (**2**)

Yield: 66% (82 mg). Orange-red single crystals were obtained by slow diffusion of *n*-hexane into acetone solution.

^1^H NMR [(CD_3_)_2_CO, Me_4_Si, δ/ppm]: 9.24 (s, 1H, C_5_H_4_CHO), 9.03 (d, 2H, ^3^*J*_HH_ = 6.5, H6), 7.74 (d, 2H, ^3^*J*_HH_ = 2.5, H3), 7.45 (m, 3H, H_*para*_PPh_3_), 7.36 (m, 6H, H_*meta*_PPh_3_), 7.11 (t, 6H, ^3^*J*_HH_ = 8.4, H_*ortho*_PPh_3_), 7.04 (dd, 2H, *J*_HH_ = 2.5; 6.5, H5), 5.71 (broad, 2H, H_β_-C_5_H_4_CHO), 4.76 (broad, 2H, H_γ_-C_5_H_4_CHO), 3.97 (s, 6H, OCH_3_). APT-^13^C{^1^H} NMR [(CD_3_)_2_CO, δ/ppm]: 189.5 (C_5_H_4_CHO), 167.8 (C2), 158.1 (C4), 157.3 (C6), 133.9 (d, ^2^*J*_CP_ = 11, CH_*ortho*_PPh_3_), 132.2 (d, ^1^*J*_CP_ = 43, C_*q*_-PPh_3_), 131.2 (d, ^4^*J*_CP_ = 2, CH_*para*_PPh_3_), 129.4 (d, ^3^*J*_CP_ = 9, CH_*meta*_PPh_3_), 113.8 (C5), 110.4 (C3), 84.5 (C_β_-C_5_H_4_CHO), 78.0 (C_γ_-C_5_H_4_CHO), 57.1 (OCH_3_). ^31^P{^1^H} NMR [(CD_3_)_2_CO, δ/ppm]: 49.5 (s, PPh_3_). FTIR [KBr, cm^–1^]: 3075–3060 (υ_C–H_ aromatic rings), 2929 (υ_C–H_ alkanes), 1670 (υ_C=O_), 1440 (υ_C=C_), 1260 (υ_CF3SO3_), 1223 (υ_C–O_). UV–vis [DMSO, λ_max_/nm (ε x 10^3^/M^–1^ cm^–1^)]: 290 (sh), 380 (7.21), 415 (sh). UV–vis [CH_2_Cl_2_, λ_max_/nm (ε x 10^3^/M^–1^ cm^–1^)]: 268 (sh), 292 (sh), 337 (sh), 393 (4.0). Elemental analysis calcd for C_37_H_32_F_3_N_2_O_6_PRuS (821.76): C, 54.08, H, 3.92; N, 3.41; S, 3.90. Found: C, 54.1; H, 4.0; N, 3.4; S, 4.0.

#### [Ru(η^5^-C_5_H_4_CH_2_OH)(4,4′-OCH_3_–2,2′-bipy)(PPh_3_)][CF_3_SO_3_] (**3**)

Yield: 50% (62 mg, method A); 71% (88 mg, method B).

^1^H NMR [(CD_3_)_2_CO, Me_4_Si, δ/ppm]: 9.18 (d, 2H, ^3^*J*_HH_ = 6.5, H6), 7.74 (d, 2H, ^3^*J*_HH_ = 2.7, H3), 7.42 (m, 3H, H_*para*_PPh_3_), 7.33 (m, 6H, ^3^*J*_HH_ = 7.2, H_*meta*_PPh_3_), 7.15 (t, 6H, ^3^*J*_HH_ = 8.2, H_*ortho*_PPh_3_), 6.96 (dd, 2H, *J*_HH_ = 2.7; 6.5, H5), 4.82 (broad, 2H, H_β_-C_5_H_4_CH_2_OH), 4.50 (m, 2H, H_γ_-C_5_H_4_CH_2_OH), 4.10 (s, 2H, C_5_H_4_CH_2_OH), 3.98 (s, 6H, OCH_3_). APT-^13^C{^1^H} NMR [(CD_3_)_2_CO, δ/ppm]: 167.2 (C2), 158.1 (C4), 157.5 (C6), 133.9 (d, ^2^*J*_CP_ = 11, CH_*ortho*_PPh_3_), 133.3 (d, ^1^*J*_CP_ = 41, C_*ipso*_PPh_3_), 130.7 (d, ^4^*J*_CP_ = 2, CH_*para*_PPh_3_), 129.3 (d, ^3^*J*_CP_ = 9, CH_*meta*_PPh_3_), 113.4 (C5), 110.0 (C3), 104.1 (d, ^2^*J*_CP_ = 6, C_α_-C_5_H_4_CH_2_OH), 75.5 (C_β_-C_5_H_4_CH_2_OH), 75.4 (d, ^2^*J*_CP_ = 2, C_γ_-C_5_H_4_CH_2_OH), 57.9 (CH_2_OH), 57.0 (OCH_3_). ^31^P NMR [(CD_3_)_2_CO, δ/ppm]: 51.9 (s, PPh_3_). FTIR [KBr, cm^–1^]: 3415 (υ_O–H_), 3071–3050 (υ_C–H_ aromatic rings), 2922 (υ_C–H_ alkanes), 1440 (υ_C=C_), 1258 (υ_CF3SO3_ counterion), 1222 (υ_C–O_). UV–vis [DMSO, λ_max_/nm (ε × 10^3^/M^–1^ cm^–1^)]: 290 (21.2), 342 (sh), 416 (3.9), 472 (sh). UV–vis [CH_2_Cl_2,_ λ_max_/nm (ε × 10^3^/M^–1^ cm^–1^)]: 271 (24.1), 295 (sh), 347 (sh), 427 (3.9), 470 (sh). Elemental analysis calcd for C_37_H_34_F_3_N_2_O_6_PRuS (823.78): C, 53.95; H, 4.16; N, 3.40; S, 3.89. Found: C, 54.2; H, 4.2; N, 3.4; S, 4.0.

### Esterification with Biotin (**4**, **5**, and **6**)

To a stirred solution of [Ru(η^5^-C_5_H_4_CH_2_OH)(4,4′-R-2,2′-bipy)(PPh_3_)][CF_3_SO_3_] (R = H 100 mg; R = CH_3_ 102 mg; R = OCH_3_ 106 mg; 0.1 mmol, respectively) and 5-[(3a*S*,4*S*,6a*R*)-2-oxohexahydro-1*H*-thieno[3,4-*d*]imidazol-4-yl]pentanoic acid (biotin) (44 mg, 0.18 mmol) in DMF (8 mL), EDC·Cl (39 mg, 0.2 mmol) and DMAP (10 mg; 0.08 mmol) were added. The orange mixture was stirred for 14 h at room temperature. After that, the solvent was removed under vacuum, and the residue obtained was washed with water (10 mL × 3) and diethyl ether (10 mL × 3) to afford the pure product as bright-orange solids.

#### [Ru(η^5^-C_5_H_4_CH_2_Biotin)(2,2′-bipy)(PPh_3_)][CF_3_SO_3_] (**4**)

Yield: 80% (79 mg).

^1^H NMR [(CD_3_)_2_CO, Me_4_Si, δ/ppm]: 9.51 (d, 2H, ^3^*J*_HH_ = 5.4, H6), 8.24 (d, 2H, ^3^*J*_HH_ = 8.0, H3), 7.94 (t, 2H, ^3^*J*_HH_ = 7.7, H4), 7.40 (comp, 5H, H_*p*_PPh_3_ + H5), 7.33 (t, 6H, ^3^*J*_HH_ = 8.0, H_*m*_-PPh_3_), 7.11 (t, 6H, ^3^*J*_HH_ = 8.0, H_*o*_-PPh_3_), 5.76 (s br, 2H, NH), 5.14 (s br, 2H, H_β_-η^5^-C_5_H_4_CH_2_Biotin), 4.62 (s br, 2H, H_γ_-η^5^-C_5_H_4_CH_2_Biotin), 4.60 (s br, 2H, η^5^-C_5_H_4_CH_2_Biotin), 4.50 (m, 1H, SCH_2_-CH^Biotin^), 4.29 (m, 1H, CH^Biotin^), 3.15 (m, 1H, S-CH^Biotin^), 2.95* + 2.72 (d, 1H, ^3^*J*_HH_ = 12.6, SCH_2_^Biotin^), 1.95 (t, 2H, ^3^*J*_HH_ = 7.2, COCH_2_^Biotin^), 1.64 + 1.45–1.29 (3 m, 6H, CH_2_CH_2_CH_2_^Biotin^). *(under the residual water peak of the solvent) APT-^13^C{^1^H} NMR [(CD_3_)_2_CO, δ/ppm]: 172.9 (CO, C_5_H_4_CH_2_Biotin ester), 163.6 (CO, η^5^-C_5_H_4_CH_2_Biotin, urea), 156.4 (C2), 156.2 (C6), 149.6 (C4), 133.9 (d, ^2^*J*_CP_ = 11, CH_*o*_*-*PPh_3_), 133.0 (d, ^1^*J*_CP_ = 41, C_*q*_*-*PPh_3_), 130.9 (d, ^4^*J*_CP_ = 2, CH_*p*_*-*PPh_3_), 129.6 (d, ^3^*J*_CP_ = 10, CH_*m*_*-*PPh_3_), 127.3 (C5), 124.8 (C3), 96.7 (d, ^2^*J*_CP_ = 8, C_α_–C_5_H_4_CH_2_Biotin), 79.4, 79.1 (d, ^2^*J*_CP_ = 2, C_γ_–C_5_H_4_CH_2_Biotin), 76.2 (C_β_–C_5_H_4_CH_2_Biotin), 62.4 (C_5_H_4_CH_2_Biotin), 60.8 (SCHCH^Biotin^), 59.8 (SCH_2_-CH^Biotin^), 56.4 (SCH^Biotin^), 40.9 (SCH_2_^Biotin^), 33.8 + 29.1* + 25.4 (CH_2_CH_2_CH_2_CH_2_^Biotin^). *(under the solvent peak).

^31^P NMR [(CD_3_)_2_CO, δ/ppm]: 50.9 (s, PPh_3_). FTIR [KBr, cm^–1^]: 3384 and 3240 (υ_N–H_), 3074–3057 (υ_C–H_ aromatic rings), 2855 (υ_C–H_ alkanes), 1730 and 1697 (υ_C=O_), 1485 (υ_C=C_ aromatic rings), 1262 (υ_CF3SO3_ counterion). UV–vis [DMSO, λ_max_/nm (ε × 10^3^/M^–1^ cm^–1^)]: 294 (19.5), 350 (sh), 414 (3.6), 488 (sh). UV–vis [CH_2_Cl_2,_ λ_max_/nm (ε × 10^3^/M^–1^ cm^–1^)]: 290 (18.4), 347 (sh), 419 (3.9), 480 (sh). Elemental analysis calcd for C_45_H_4_F_3_N_4_O_6_PRuS_2_ (990.02): C, 54.59; H, 4.48; N, 5.66; S, 6.48. Found: C, 54.7; H, 4.5; N, 5.8; S, 7.0.

#### [Ru(η^5^-C_5_H_4_CH_2_Biotin)(4,4′-CH_3_–2,2′-bipy)(PPh_3_)][CF_3_SO_3_] (**5**)

Yield: 71% (72 mg).

^1^H NMR [(CD_3_)_2_CO, Me_4_Si, δ/ppm]: 9.32 (d, 2H, ^3^*J*_HH_ = 5.8, H6), 8.08 (s, 2H, H3), 7.42 (t, 3H, ^3^*J*_HH_ = 7.0, H_*para*_PPh_3_), 7.32 (t, 6H, ^3^*J*_HH_ = 7.2, H_*meta*_PPh_3_), 7.23 (d, 2H, ^3^*J*_HH_ = 5.7, H5), 7.12 (t, 6H, ^3^*J*_HH_ = 8.2, H_*ortho*_PPh_3_), 5.69 (br s, 1H, NH), 5.63 (br s, 1H, NH), 5.10 (s, 2H, H_β_-C_5_H_4_CH_2_Biotin), 4.59 (s, 2H, H_γ_-C_5_H_4_CH_2_Biotin), 4.61 (s, 2H, C_5_H_4_CH_2_Biotin), 4.51 (m, 1H, SCH_2_CH^Biotin^), 4.31 (m, 1H, CH^Biotin^), 3.17 (m, 1H, SCH^Biotin^), 2.49 (s, 6H, CH_3_), 2.92 (m, 2H, SCH_2_^Biotin^), 1.66-1.47 (3 m, 6H, CH_2_CH_2_CH_2_^Biotin^). APT-^13^C{^1^H} NMR [(CD_3_)_2_CO, δ/ppm]: 172.9 (CO, C_5_H_4_CH_2_Biotin ester), 163.6 (CO, η^5^-C_5_H_4_CH_2_Biotin, urea), 156.4 (C2), 156.2 (C6), 149.6 (C4), 133.9 (d, ^2^*J*_CP_ = 11, CH_*o*_*-*PPh_3_), 133.0 (d, ^1^*J*_CP_ = 41, C_*q*_*-*PPh_3_), 130.9 (d, ^4^*J*_CP_ = 2, CH_*p*_*-*PPh_3_), 129.6 (d, ^3^*J*_CP_ = 10, CH_*m*_*-*PPh_3_), 127.3 (C5), 124.8 (C3), 95.8 (d, ^2^*J*_CP_ = 8, C_α_-C_5_H_4_CH_2_Biotin), 79.1 (2 s, C_γ_-C_5_H_4_CH_2_Biotin), 76.2 (2 s, C_β_-C_5_H_4_CH_2_Biotin), 62.4 (C_5_H_4_CH_2_Biotin), 60.8 (SCHCH^Biotin^), 60.0 (SCH_2_-CH^Biotin^), 56.4 (SCH^Biotin^), 41.0 (SCH_2_^Biotin^), 33.9 + 29.1* + 25.4 (CH_2_CH_2_CH_2_CH_2_^Biotin^), 20.9 (CH_3_). *(under the solvent peak) ^31^P NMR [(CD_3_)_2_CO, δ/ppm]: 50.8 (s, PPh_3_). FTIR [KBr, cm^–1^]: 3350 and 3241 (υ_N–H_), 3059 (υ_C–H_ aromatic rings), 2889 (υ_C–H_ alkanes), 1731–1699 (υ_C=O_), 1440 (υ_C=C_ aromatic rings), 1260 (υ_CF3SO3_ counterion). UV–vis [DMSO, λ_max_/nm (ε × 10^3^/M^–1^ cm^–1^)]: 294 (20.3), 356 (sh), 406 (3.6), 478 (sh). UV–vis [CH_2_Cl_2,_ λ_max_/nm (ε × 10^3^/M^–1^ cm^–1^)]: 242 (sh), 288 (22.1), 341 (sh), 412 (4.3), 473 (sh). Elemental analysis calcd for C_47_H_48_F_3_N_4_O_6_PRuS_2_ (1018.08): C, 55.45; H, 4.75; N, 5.50; S, 6.30. Found: C, 55.2; H, 4.8; N, 5.4; S, 6.0.

#### [Ru(η^5^-C_5_H_4_CH_2_Biotin)(4,4′-OCH_3_–2,2′-bipy)(PPh_3_)][CF_3_SO_3_] (**6**)

Yield: 77% (81 mg).

^1^H NMR [(CD_3_)_2_CO, Me_4_Si, δ/ppm]: 9.19 (d, 2H, ^3^*J*_HH_ = 6.4, H6), 7.80 (m, 2H, H3), 7.42 (t, 3H, ^3^*J*_HH_ = 7.0, H_*para*_PPh_3_), 7.33 (t, 6H, ^3^*J*_HH_ = 7.2, H_*meta*_PPh_3_), 7.15 (t, 6H, ^3^*J*_HH_ = 8.2, H_*ortho*_PPh_3_), 6.96 (dd, 2H, *J*_HH_ = 2.7; 6.5, H5), 5.66 (broad s, 1H, NH), 5,60 (broad s, 1H, NH), 5.03 (s, 2H, H_β_-C_5_H_4_CH_2_Biotin), 4.58 (s, 2H, C_5_H_4_CH_2_Biotin), 4.48 (comp, 3H, H_γ_-C_5_H_4_CH_2_Biotin + SCH_2_-CH^Biotin^), 4.30 (m, 1H, CH^Biotin^), 3.99 (s, 6H, OCH_3_), 3.16 (m, 1H, S-CH^Biotin^), 2.49 (s, 6H, CH_3_), 2.92 (m, 2H, SCH_2_^Biotina^), 1.66-1.47 (4 m, 8H, CH_2_CH_2_CH_2_^Biotina^). APT-^13^C{^1^H} NMR [(CD_3_)_2_CO, δ/ppm]: 173.1 (CO, C_5_H_4_CH_2_Biotin ester), 167.4 (C2), 163.8 (CO, η^5^-C_5_H_4_CH_2_Biotin, urea), 158.1 (C6), 157.4 (C4), 133.9 (d, ^2^*J*_CP_ = 11, CH_*ortho*_PPh_3_), 133.1 (d, ^1^*J*_CP_ = 41, C_*ipso*_PPh_3_), 130.8 (d, ^4^*J*_CP_ = 2, C H_*para*_PPh_3_), 129.3 (d, ^3^*J*_CP_ = 9, CH_*meta*_PPh_3_), 113.6 (2 s, C5), 110.1 (2 s, C3), 95.3 (2 s, C_α_-C_5_H_4_CH_2_OH), 78.7 (C_β_-C_5_H_4_CH_2_OH), 75.3 (2 s, C_γ_-C_5_H_4_CH_2_OH), 62.4 (C_5_H_4_CH_2_Biotin), 60.8 (SCHCH^Biotin^), 60.1 (SCH_2_-CH^Biotin^), 57.1 (OCH_3_), 56.4 (SCH^Biotin^), 41.0 (SCH_2_^Biotin^), 34.0 + 29.1* + 25.4 (CH_2_CH_2_CH_2_CH_2_^Biotin^). *(under the solvent peak) ^31^P NMR [(CD_3_)_2_CO, δ/ppm]: 51.5 (s, PPh_3_). FTIR [KBr, cm^–1^]: 3240 (υ_N–H_), 3071–3057 (υ_C–H_ aromatic rings), 2860 (υ_C–H_ alkanes), 1731 and 1695 (υ_C=O_), 1495 (υ_C=C_ aromatic rings), 1260 (υ_CF3SO3_ counterion), 1220 (υ_C–O_). UV–vis [DMSO, λ_max_/nm (ε × 10^3^/M^–1^ cm^–1^)]: 294 (20.5), 350 (sh), 414 (3.2), 480 (sh). UV–vis [CH_2_Cl_2_, λ_max_/nm (ε × 10^3^/M^–1^ cm^–1^)]: 273 (21.3), 345 (sh), 422 (3.7), 472 (sh). Elemental analysis calcd for C_47_H_48_F_3_N_4_O_8_PRuS_2_ (1500.07): C, 53.76; H, 4.61; N, 5.34; S, 6.11. Found: C, 53.9; H, 4.6; N, 5.1; S, 6.6.

### X-ray Structure Analysis

The X-ray intensity data were measured on a D8 QUEST ECO three-circle diffractometer system equipped with a PHOTON II CMOS detector, a ceramic x-ray tube (Mo Kα, λ = 0.71076 Å), and a doubly curved silicon crystal Bruker Triumph monochromator.^29^ Measurements were performed at low temperatures (100 K). The frames were integrated with the Bruker SAINT software package using a narrow-frame algorithm.^30^ The structure was solved and refined using the Bruker SHELXTL Software Package.^31^

The crystallographic data and details of the structure solution and refinement procedures are reported in the Supporting Information.

### Stability Studies

For the stability studies, all complexes were first dissolved in 100% DMSO, and a sample containing each compound in 2% DMSO/DMEM at 100–150 μM was prepared. For each compound, their electronic spectrum was recorded in the range allowed by the solvent mixture at set time intervals. Samples were stored at room temperature and protected from light between measurements. The relative absorbance variation (% variation) was calculated between measurements with the following expression (*t*_mix_ indicates the time of the first data record, immediately after dissolution)
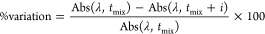
A % variation below ≤10% over 24 h (associated with maintenance of >∼90% of the parent compound in solution) is considered adequate for biological evaluation.

### Cell Studies

#### Cell Lines

Human NSCLC cells A549, NCI-H2228, Calu-3, NCI-H1975, and murine kidney MDCK cells were from ATCC (Manassas, VA). MDCK-P-gp cells, stably overexpressing this transporter, were a kind gift of Dr. Marialessandra Contino, Department of Pharmacy, University of Bari, and are described in ref (32). Cells were grown in RPMI-1640 medium, supplemented with 10% v/v FBS and 1% penicillin–streptomycin, at 37 °C, 5% CO_2_, in a humidified atmosphere.

#### Cytotoxic Activity

Cells were seeded in 96 well plates. Compounds were first dissolved in DMSO to a 10 mM stock solution and then further diluted in a growth medium (DMSO concentration <1%). Untreated cells (control cells) were incubated with 1% DMSO as a vehicle. In the first experimental set, cells were incubated for 72 h at the following concentrations: 1, 10, and 100 nM and 1, 10, and 100 μM. In a second experimental set, cells were incubated with cisplatin at the following concentrations: 1, 10, and 100 nM; 1, 10, and 100 μM; and alone or in the presence of 0.1 μM of the indicated compounds. In the third experimental set, cells were incubated for 72 h with 1 μM of the selected compound plus cisplatin at the following concentrations: 1, 10, and 100 nM and 1, 10, and 100 μM. Cell viability was evaluated using the WST-1 assay (Sigma-Merck), as per the manufacturer’s instructions, using a Packard EL340 microplate reader (Bio-Tek Instruments, Winooski, VT). The absorbance units of the untreated cells were considered 100%; the absorbance units of the other experimental conditions were expressed as percentages versus untreated cells. IC_50,_ defined as the concentration of the compound, cisplatin, or their combinations that killed 50% of cells, was calculated using the GraphPrism software (v9).

#### ATPase Activity

The P-gp ATPase activity was measured in membrane vesicles as detailed extensively in our previous work.^15^ The absorbance of the phosphate hydrolyzed from ATP was measured at 620 nm, using a Packard EL340 microplate reader, and transformed into nmoles hydrolyzed phosphate (Pi)/min/mg proteins, according to a titration curve previously prepared.

#### Site-Directed Mutagenesis

The pHa vector containing the full-length mdr1/P-gp cDNA (Addgene, Cambridge, MA) was subcloned into a pCDNA3 vector (Addgene) and sequenced to verify the wild-type sequence of mdr1/P-gp. The P-gp-expressing pCDNA3 vector was subjected to PCR-based site-specific mutagenesis using the QuikChange kit (Stratagene, La Jolla, CA), following the manufacturer’s instructions to generate the mutant constructs of P-gp. The mutations were confirmed by DNA sequencing.^33^ 5 × 10^4^ MDCK cells were seeded in FBS-free medium and treated with 3 μg mutated P-gp in 6 μL of jetPEI transfection reagent (Polyplus-transfection SA BIOPARC, Illkirch, France). After 6 h, cells were washed and grown in a complete medium for 24 h prior to their use. MDCK-P-gp cells containing wild-type P-gp were used as an internal control. The P-gp ATPase activity was measured as reported in the previous paragraph.

#### Statistical Analysis

All data in the text and figures are provided as means ± SD. The results were analyzed by one-way analysis of variance (ANOVA) and Tukey’s test. *p* < 0.05 was considered significant.

### Computational Methods

#### P-gp and Ligand Structure Set Up

To study P-gp inhibition, it is crucial to have an apo structure of the P-gp. A single structure of the*Homo Sapiens* apo P-gp protein can be found in Protein Data Bank (6FN4);^34^ however, its conformation has a closed binding pocket due to being in complex with the *Mus musculus* UIC2 Fab fragment. To overcome this problem, the AlphaFoldDB P-gp structure (P08183)^28^ was used after careful alignment and visual evaluation against experimental P-gp structures. All docked compounds (**PMC79**, **RT118**, **RT150**, **RT151**, **2**, **3**, **TM102**, **TM34**) had their structure optimized using Quantum Mechanics calculations. The Gaussian 16 software package was used,^35^ and B3LYP/6–31G* was chosen as the level of theory and the basis sets.^36−38^ The geometries obtained from the QM optimization step were very similar to the experimental crystal structures.

#### An Ala-Scanning Protocol to Identify Key P-gp Mutations

We used an *in silico* Alanine scanning protocol to identify the P-gp residues that significantly impact substrate binding. We performed this analysis using the **RT151** compound, which is an inhibitor of P-gp. Three lists of mutated residues were created corresponding to each of the P-gp binding sites (Table S6 of the SI). The mutations to alanine were performed using the mutagenesis wizard of PyMOL.^39^ Although the experiments were carried out using leucine mutations, we opted for alanine in the computational protocol due to the isotropic nature of its side chain that avoids the uncertainty associated with its conformational space.

#### Molecular Docking Settings

In previous molecular docking protocols using the P-glycoprotein,^40^ it was found that, given the lipophilic nature of the active site, Autodock Vina^41^ was the docking software that better matched experimental data. Hence, all docking calculations in this work were done using Autodock Vina 1.2.^42^ Three boxes (R-site, M-site, and H-site) were used in our protocol, each encompassing the residues of the three substrate-binding sites introduced in Ferreira et al.^43^ (Figure 8 and Table S6 of the SI). Each docking box was created using a spacing of 1.0 Å, the Autodock Vina v1.2 standard, with sizes (20,30,30), (24,30,28), and (26,28,32) and centered at (−10.466,–5.677,–27.858), (−3.294,–1.928,–49.003), and (3.456,7.964,–33.394), for the R, M, and H-site, respectively. The maximum number of binding modes and the search exhaustiveness were set to 20. All input files were prepared using AutoDockTools4,^41^ using Kollman charges^44^ on the protein and Gasteiger charges^45^ on the ruthenium-based complexes. Since ADT4 does not assign correctly the charges for buried atoms (ruthenium and phosphorous in our case), we have used the Mulliken charges (+0.38 and +0.6 for Ru and P, respectively),^46,47^ from the **TM34** compound QM optimization performed previously.^48^

## Data Availability

The authors will release the atomic coordinates upon article publication.

## Supporting Information Available

Schemes for the synthesis of compounds **1**–**6**; characterization of compounds **1**–**6** (NMR spectra and UV–vis data); crystallographic data and structural refinement details for X-ray data for **1**, **2**, and **3**; stability plots for compounds **2**–**6** in DMSO/DMEM; lists and graphical representations of the key residues constituting the P-gp binding pockets; molecular docking binding energies for an inhibitor (**RT151**) and a non-inhibitor (**RT118**) in the different pockets of the ala-mutated P-gp variants; HPLC traces (PDF)
Molecular formula strings (CSV)
PDB coordinate file (PDB)

## Supplementary Material

jm3c01120_si_002.csv

jm3c01120_si_003.pdb

## Accession Codes

CCDC 2251236 (for **1**), 2251237 (for **2**), and 2251235 (for **3**) contain supplementary crystallographic data for this paper. These data can be obtained free of charge from The Cambridge Crystallographic Data Center via www.ccdc.cam.ac.uk/products/csd/request/.

## Abbreviations Used

ABC: ATP-binding cassette
ATPase: adenosine triphosphatase
bipy: 2,2′-bipyridine
cDNA: complementary deoxyribonucleic acid
CisPt: cisplatin
Cp: cyclopentadienyl
CS: collateral sensitivity
DMAP: 4-(*N*,*N*-dimethylamino)pyridine
DMEM: Dulbecco’s modified Eagle’s medium
DMF: dimethylformamide
DMSO: dimethyl sulfoxide
EDC·Cl: 1-ethyl-3-(3-dimethylaminopropyl)carbodiimide hydrochloride
e.g.,: for example (exempli gratia)
ESI: electrospray ionization
FBS: fetal bovine serum
HMBC: heteronuclear multiple bond correlation
HMQC: heteronuclear multiple quantum correlation
HPLC: high-performance liquid chromatography
MD: molecular docking
MDR: multidrug resistance
Me: methyl
MeO: methoxy
MRP1: multidrug resistance-associated protein 1
MW: molecular weight
NMR: nuclear magnetic resonance
NSCLC: non-small cell lung cancer
P-gp: *P*-glycoprotein
TMS: tetramethylsilane
UV: ultraviolet
v/v: volume per unit of volume
Vis: visible
WST-1: sodium 4-(2-(4-iodophenyl)-3-(4-nitrophenyl)-2*H*-tetrazol-3-ium-5-yl)benzene-1,3-disulfonate
WT: wild type
