# Supplementary material for: Fighting Multidrug Resistance with Ruthenium–Cyclopentadienyl Compounds: Unveiling the Mechanism of P-gp Inhibition
Source: J Med Chem. 2023 Aug 24;66(20):14080–94. doi: 10.1021/acs.jmedchem.3c01120 (PMC10614197; doi:10.1021/acs.jmedchem.3c01120)
Supplement: Supplementary file 1 — jm3c01120_si_001.pdf [file jm3c01120_si_001.pdf]

Supplementary Information

Fighting Multidrug Resistance with Ruthenium–Cyclopentadienyl Compounds: Unveiling the Mechanism of P-gp Inhibition

Ricardo G. Teixeira,[a] Iris C. Salaroglio,[b] Nuno F. B. Oliveira,[c] João G. N. Sequeira,[c] Xavier Fontrodona,[d] Isabel Romero,[d] Miguel Machuqueiro,[c] Ana Isabel Tomaz,[a] M. Helena Garcia,[a] Chiara Riganti,*[,b,e] and Andreia Valente*[,a]

[a]Centro de Química Estrutural, Institute of Molecular Sciences and Departamento de Química e Bioquímica, Faculdade de Ciências, Universidade de Lisboa, Campo Grande, 1749-016 Lisboa, Portugal.

[b]Department of Oncology, University of Torino, 10126 Torino, Italy.

[c]BioISI: Biosystems and Integrative Sciences Institute, Faculdade de Ciências, Universidade de Lisboa, 1749-016 Lisboa, Portugal.

[d]Departament de Química and Serveis Tècnics de Recerca, Universitat de Girona, C/ M. Aurèlia Campmany, 69, E-17003 Girona, Spain.

[e]Molecular Biotechnology Center "Guido Tarone", University of Torino, 10126 Torino, Italy.

Corresponding authors

*E-mail: amvalente@ciencias.ulisboa.pt (A.Valente)

chiara.riganti@unito.it (C. Riganti)

Contents

NMR spectra S4

Electronic data S13

X-ray crystallographic structure determination S13

Stability studies in aqueous solution S21

Molecular docking studies S23

HPLC analysis S27

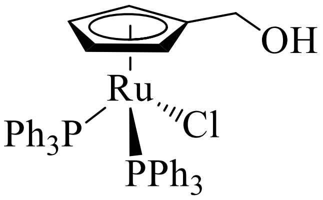

(1)

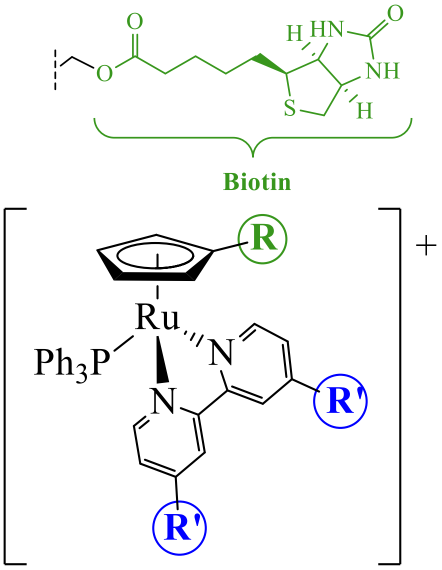

	R	R'		Ref.
**This work**	R = CHO	R' = OCH_3	**(2)**	
	R = CH_2OH	R' = OCH_3	**(3)**	
	R = CH_2Biotin	R' = H	**(4)**	
	R = CH_2Biotin	R' = CH_3	**(5)**	
	R = CH_2Biotin	R' = OCH_3	**(6)**	
Previous work	R = H	R' = CH_3	**TM102**	[a]
	R = CH_3	R' = CH_3	**RT11**	[b]
	R = CHO	R' = CH_3	**RT150**	[c]
	R = CH_2OH	R' = H	**RT118**	[c]
	R = CH_2OH	R' = CH_3	**RT151**	[c]

Scheme S1. Structurally related Ru-cyclopentadienyl compounds bearing bidentate *N*,*N*-heteroaromatic co-ligands selected for the present structure-activity study.

NMR spectra

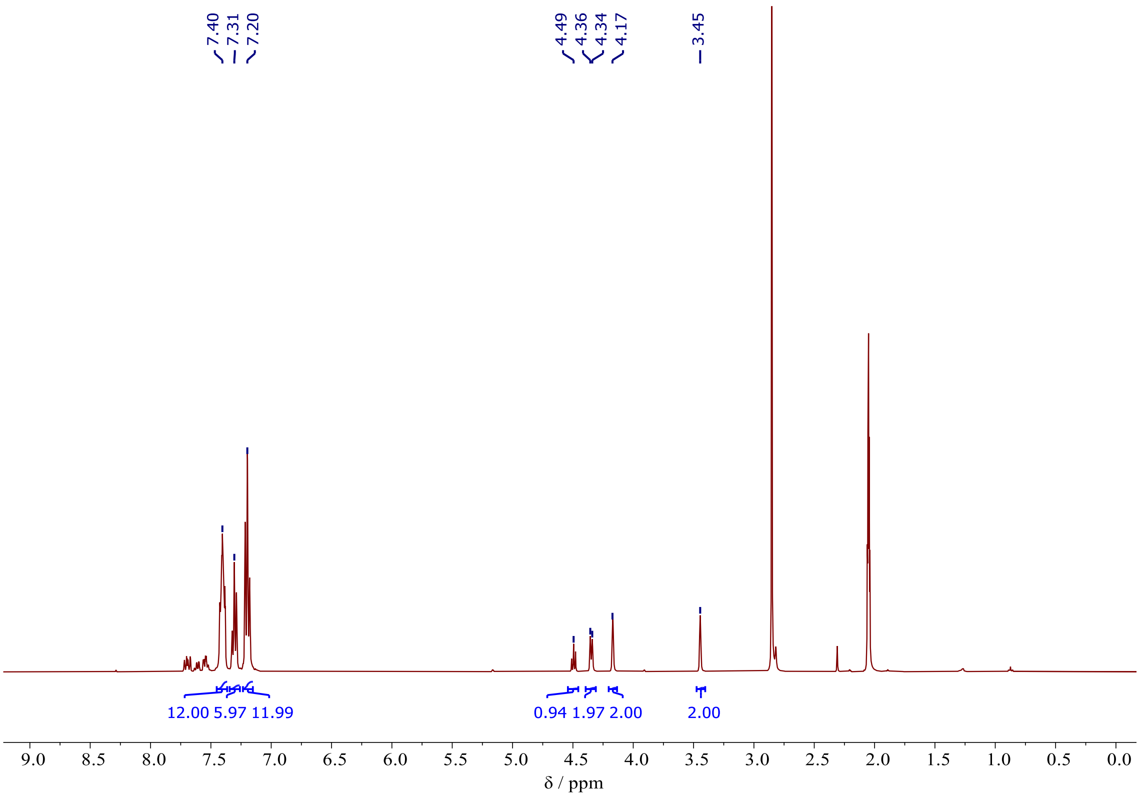

Figure S1. ^{1}H-NMR spectrum of compound **1** in acetone-d_6 at 298 K.

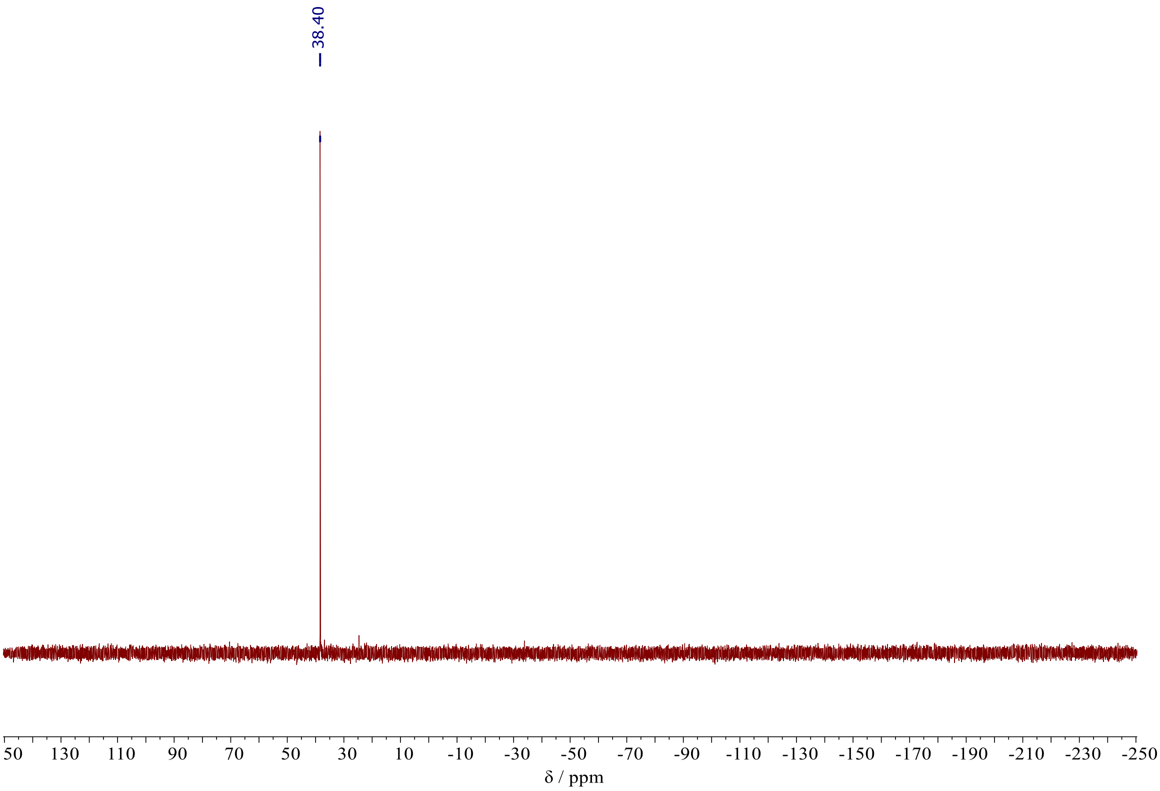

Figure S2. ^{31}P{^{1}H}-NMR spectrum of compound **1** in acetone-d_6 at 298 K.

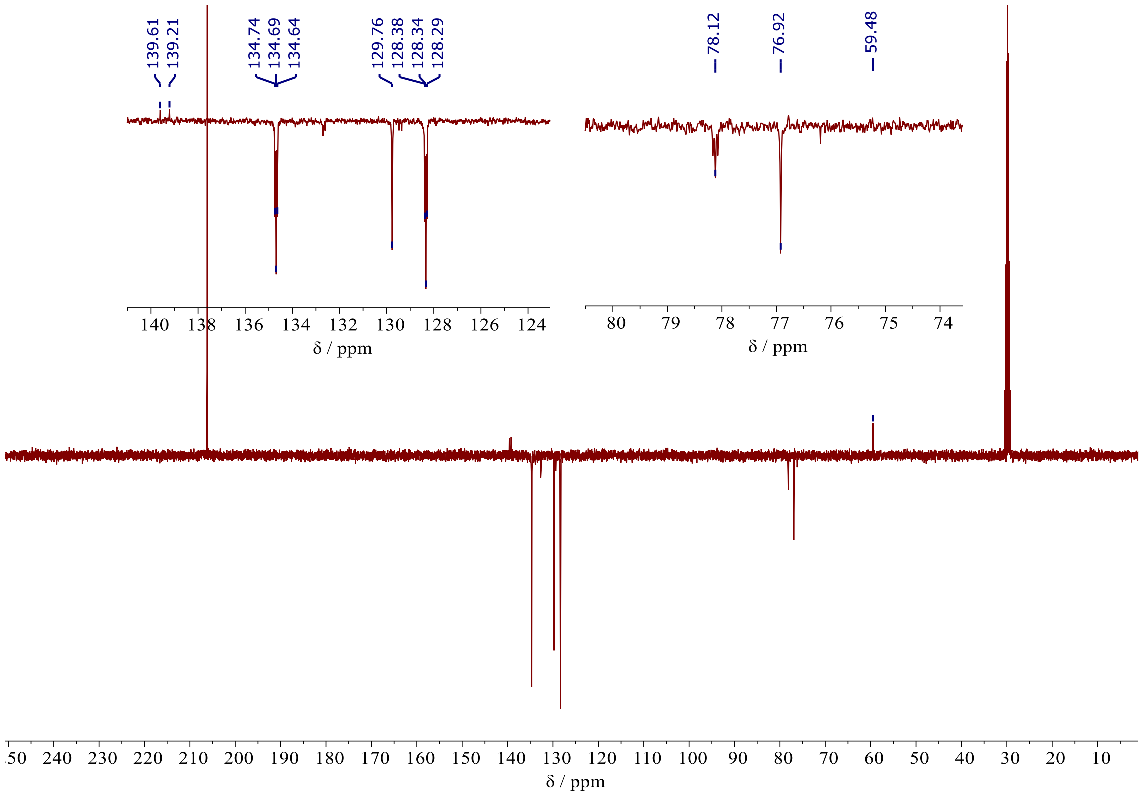

Figure S3. APT $^{13}C\{^{1}H\}$-NMR spectrum of compound **1** in acetone-d_6 at 298 K.

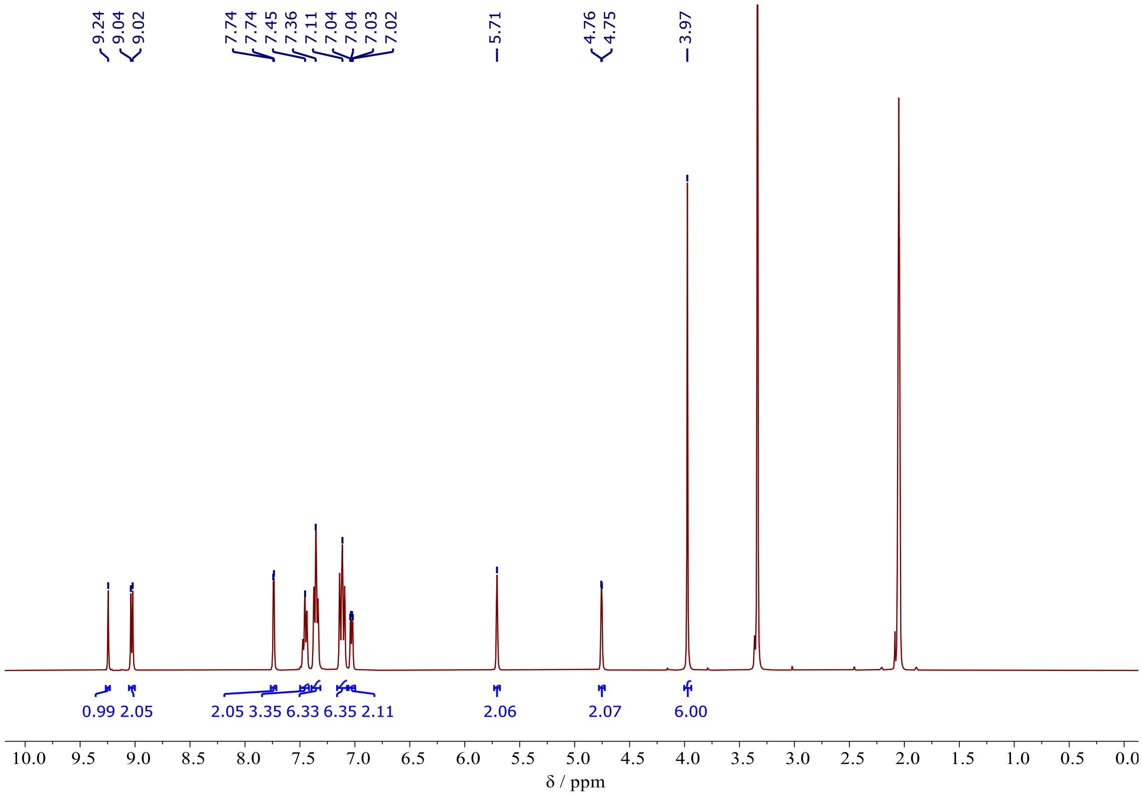

Figure S4. ^{1}H-NMR spectrum of compound **2** in acetone-d_6 at 298 K.

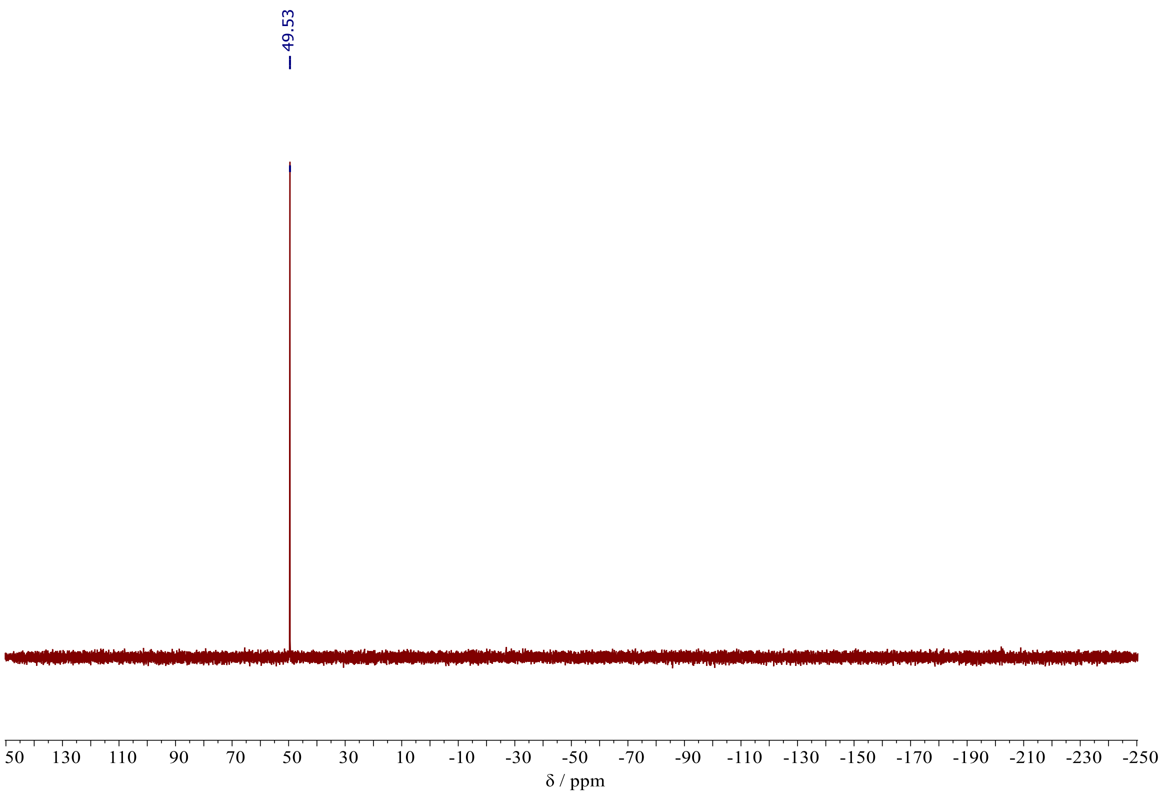

Figure S5. $^{31}P\{^{1}H\}$-NMR spectrum of compound **2** in acetone-d_6 at 298 K.

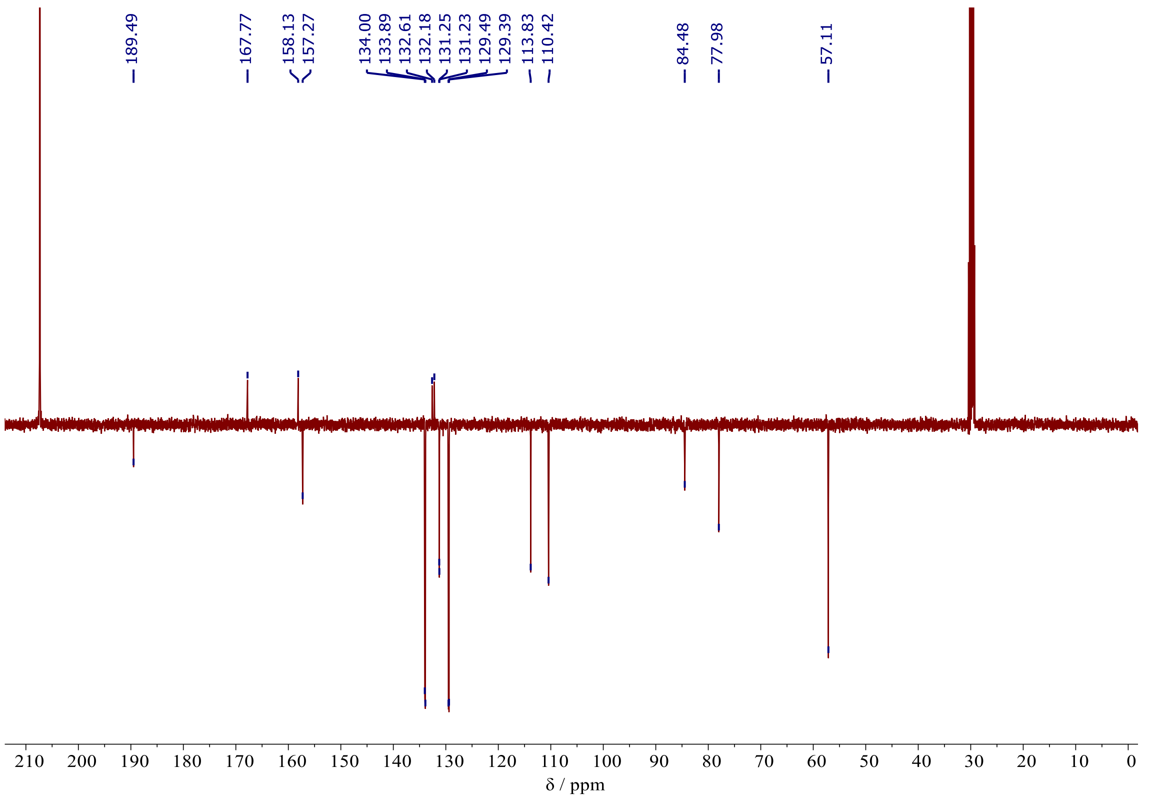

Figure S6. APT $^{13}C\{^{1}H\}$-NMR spectrum of compound **2** in acetone-d_6 at 298 K.

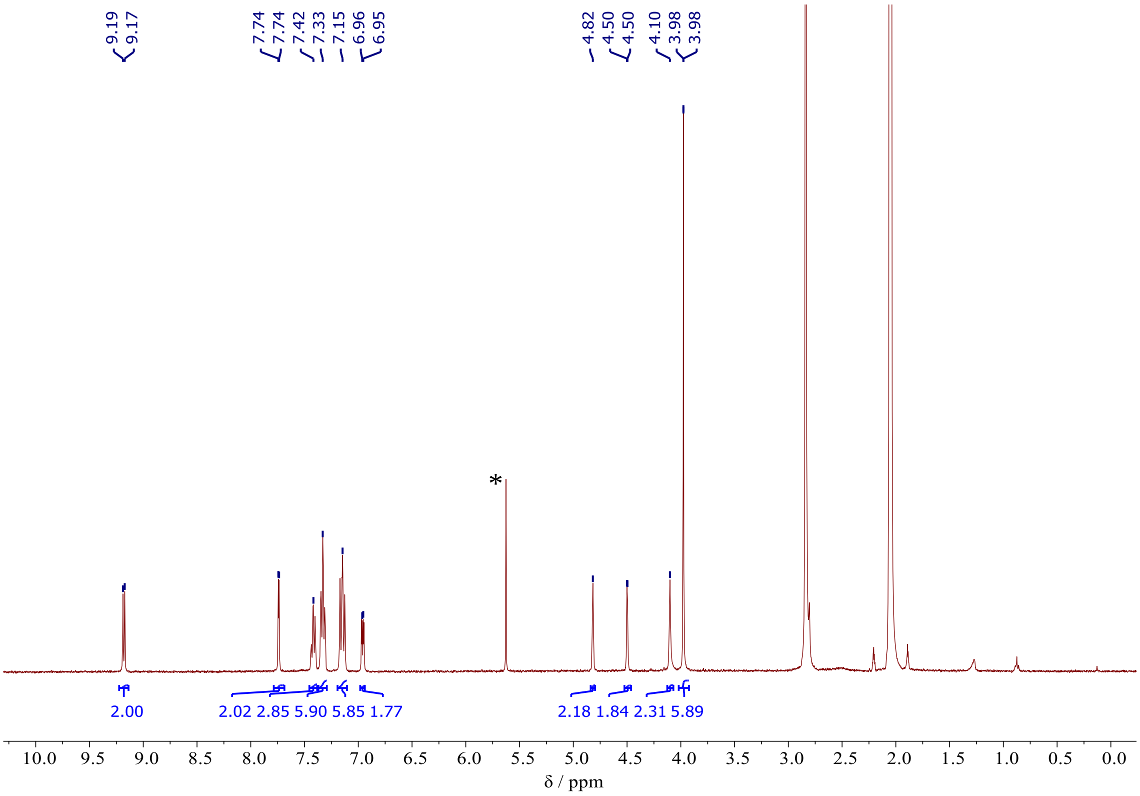

Figure S7. ^{1}H-NMR spectrum of compound **3** in acetone-d_6 at 298 K. *Residual peak of dichloromethane.

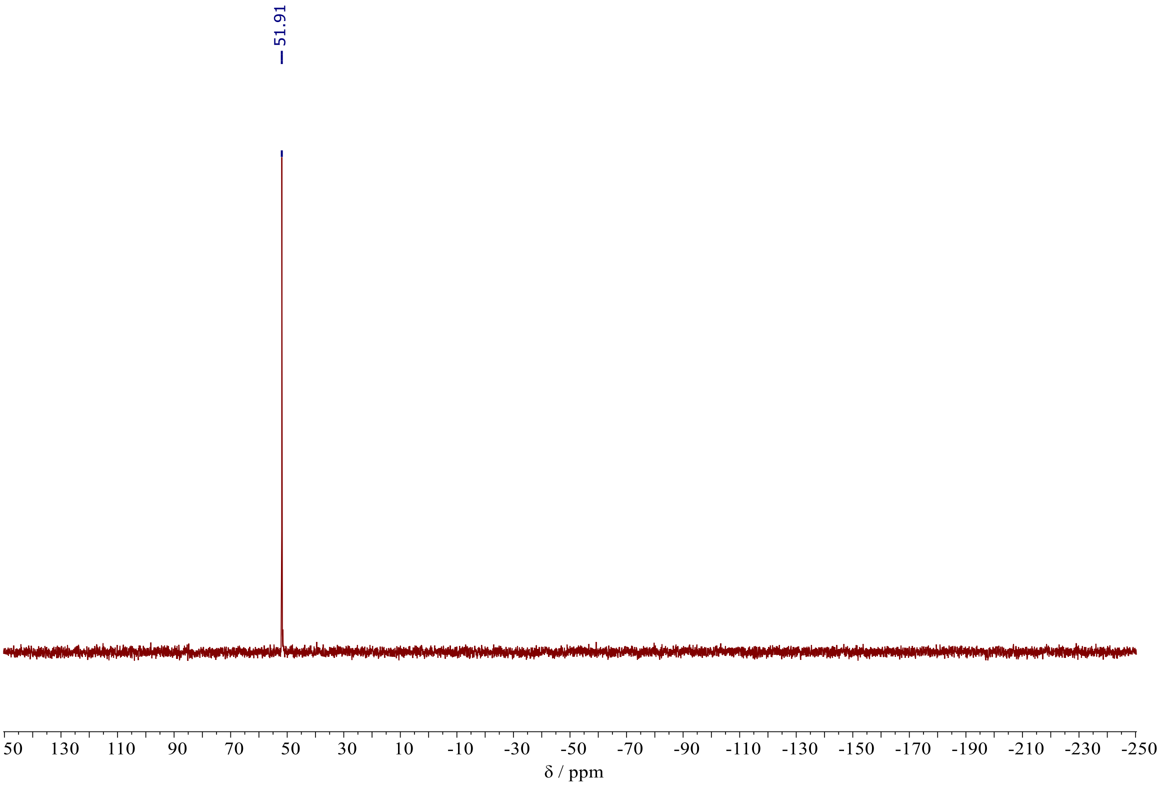

Figure S8. $^{31}P\{^{1}H\}$-NMR spectrum of compound **3** in acetone-d_6 at 298 K.

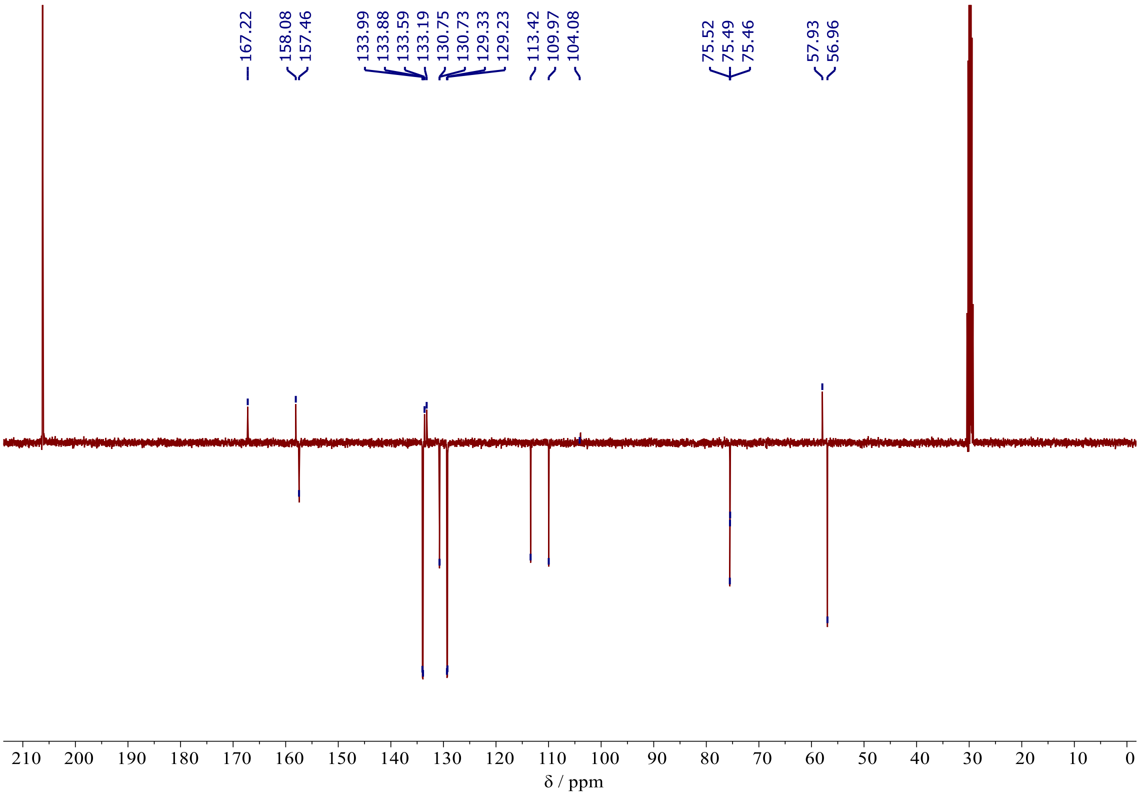

Figure S9. APT $^{13}C\{^1H\}$-NMR spectrum of compound **3** in acetone-d_6 at 298 K.

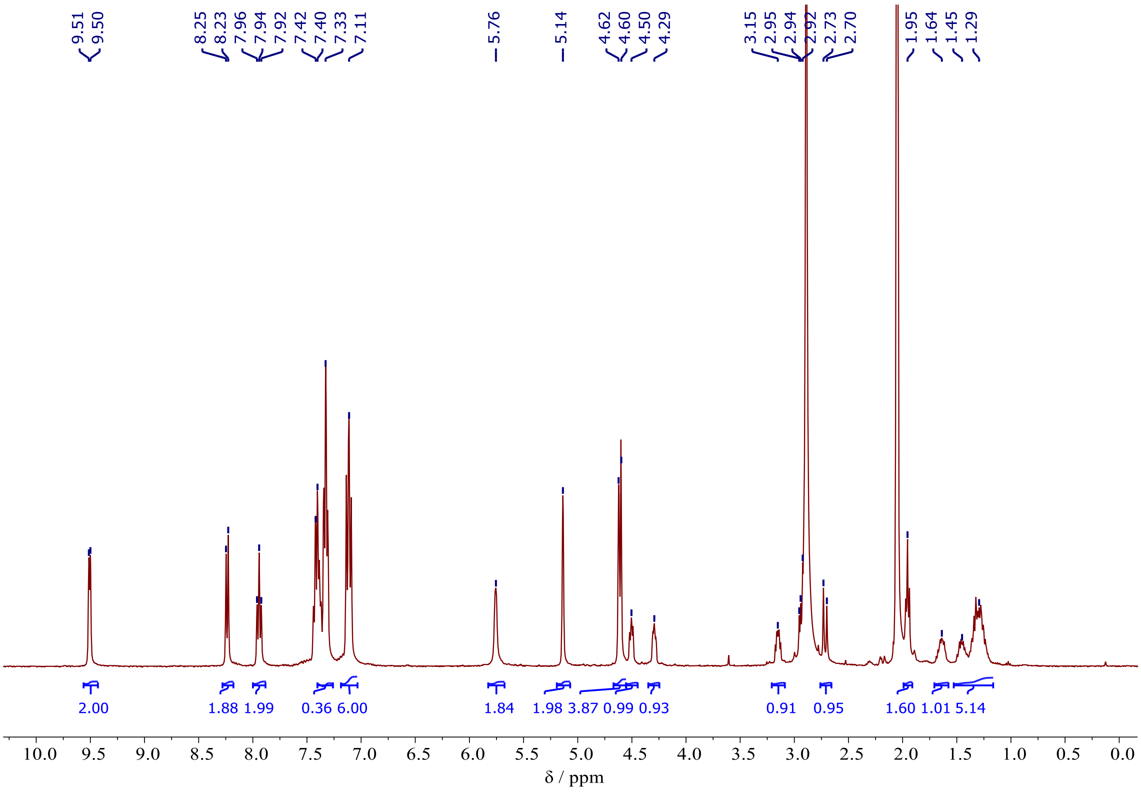

Figure S10. ^1H-NMR spectrum of compound **4** in acetone-d_6 at 298 K.

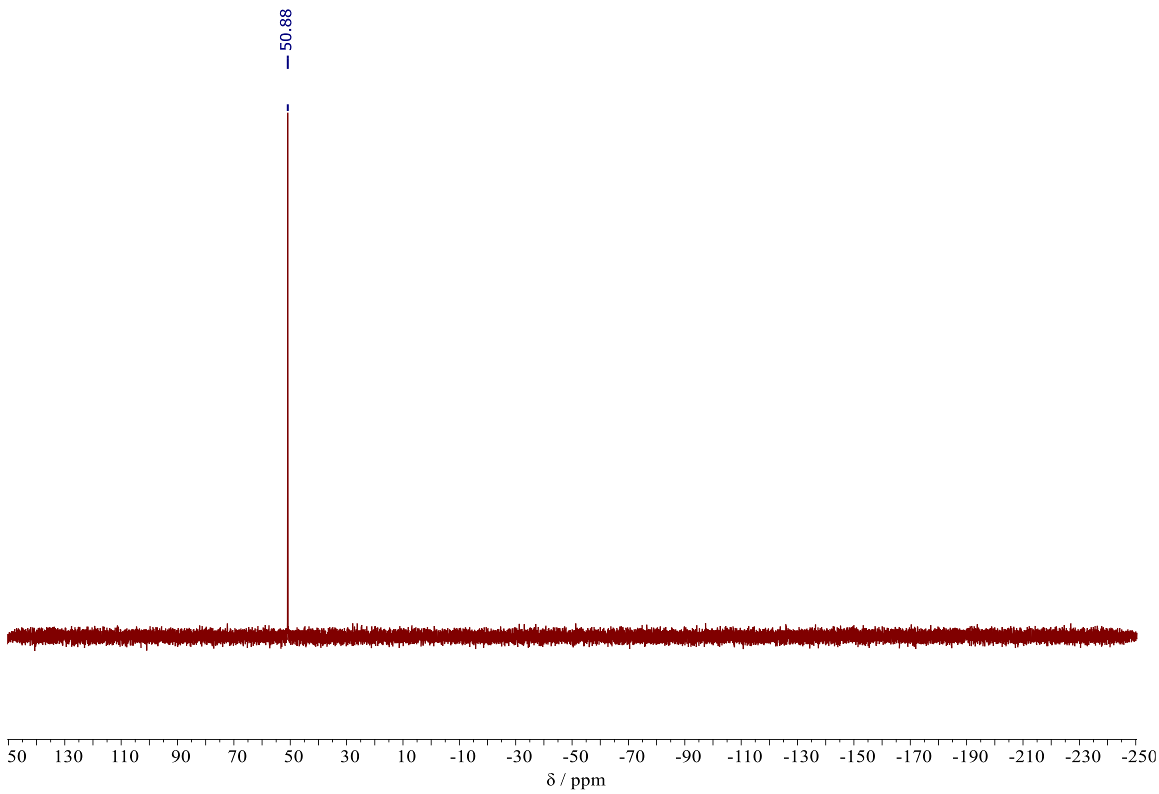

Figure S11. $^{31}P\{^{1}H\}$-NMR spectrum of compound **4** in acetone-d_6 at 298 K.

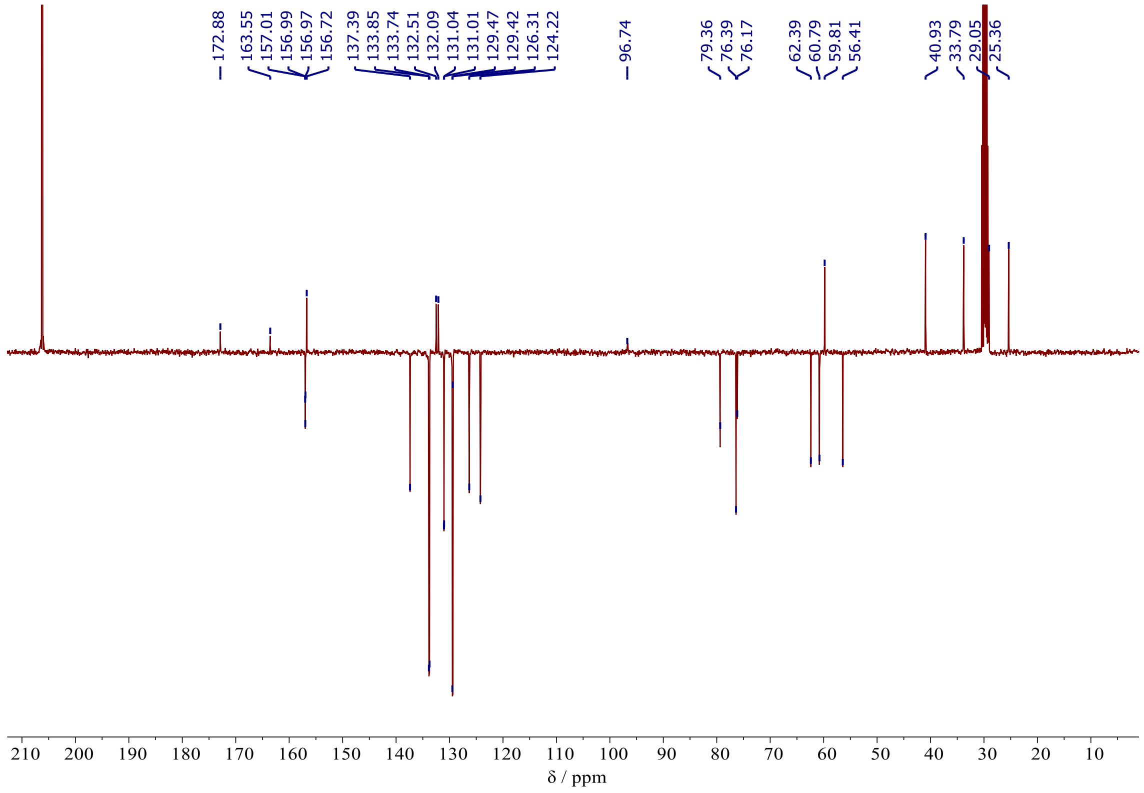

Figure S12. APT $^{13}C\{^{1}H\}$-NMR spectrum of compound **4** in acetone-d_6 at 298 K.

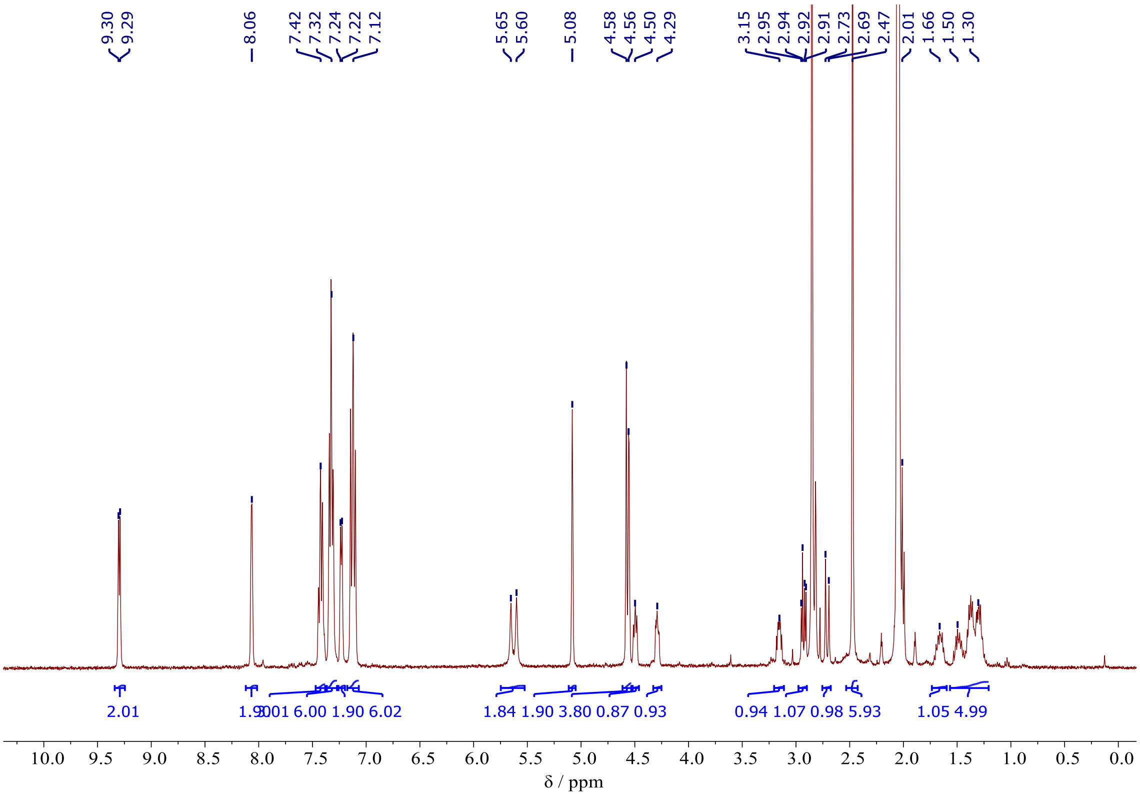

Figure S13. ^{1}H-NMR spectrum of compound **5** in acetone-d_6 at 298 K.

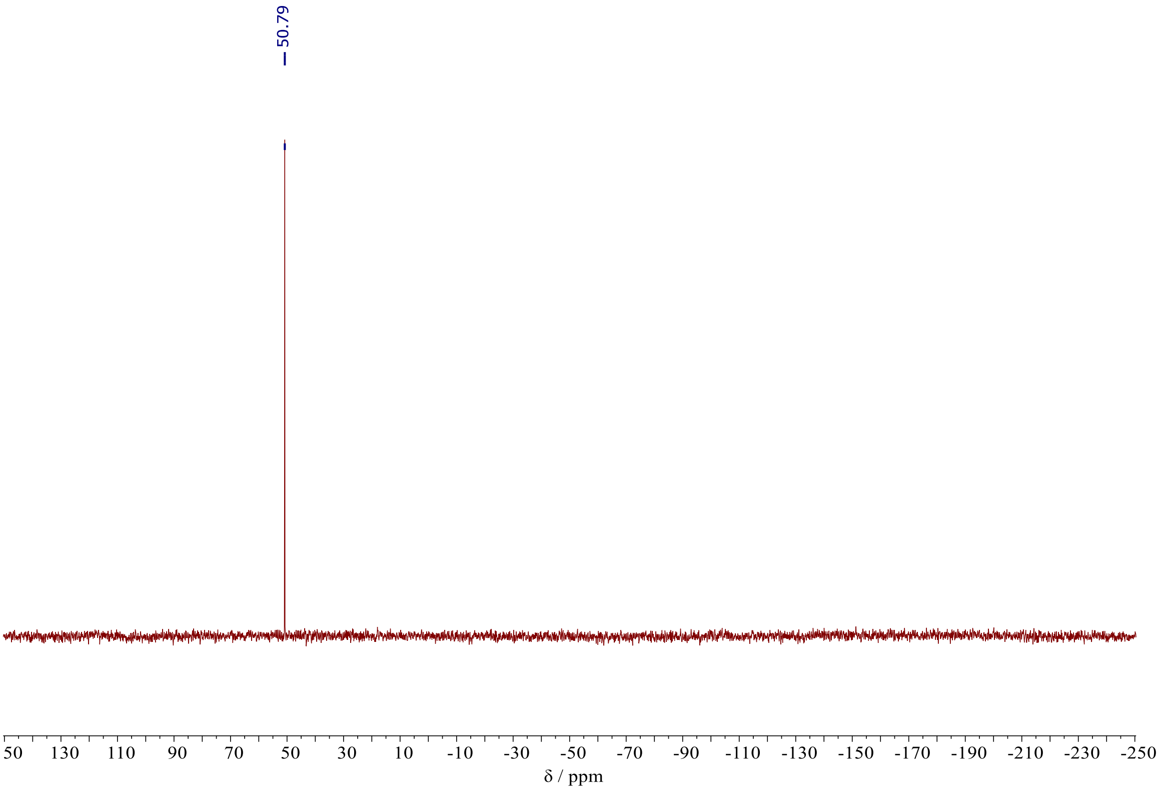

Figure S14. ^{31}P{^{1}H}-NMR spectrum of compound **5** in acetone-d_6 at 298 K.

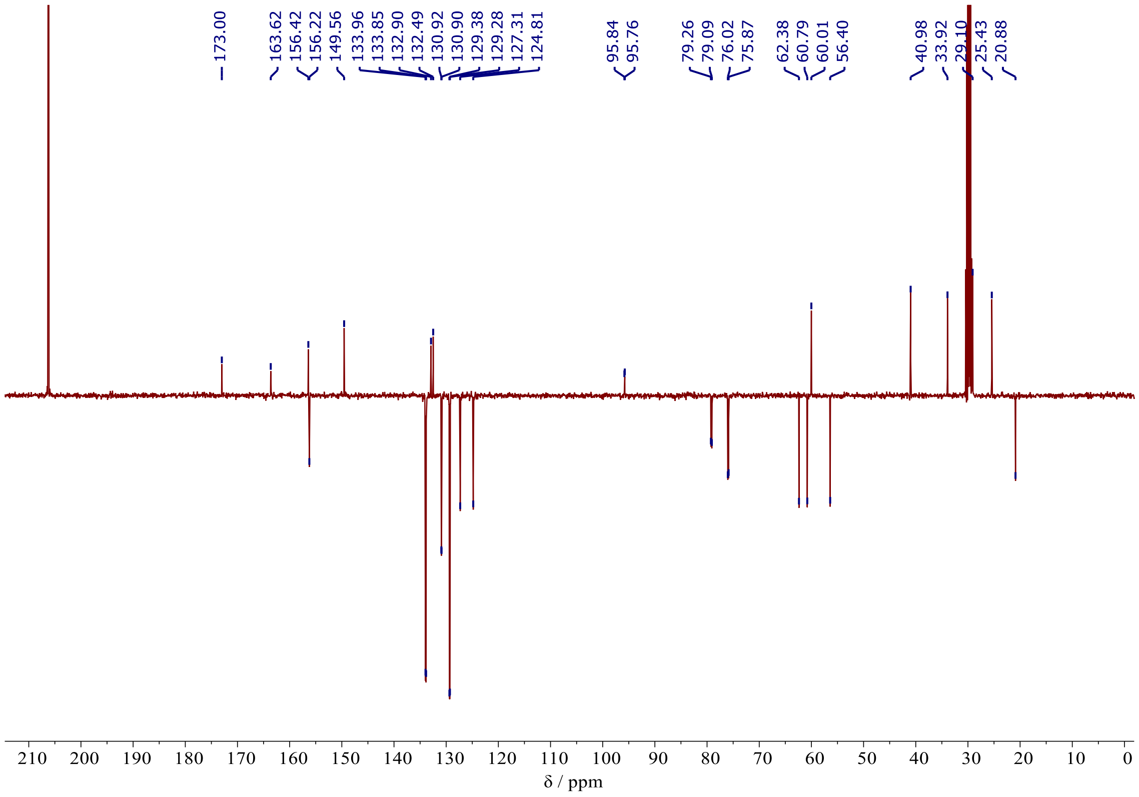

Figure S15. APT $^{13}C\{^{1}H\}$-NMR spectrum of compound **5** in acetone-d_6 at 298 K.

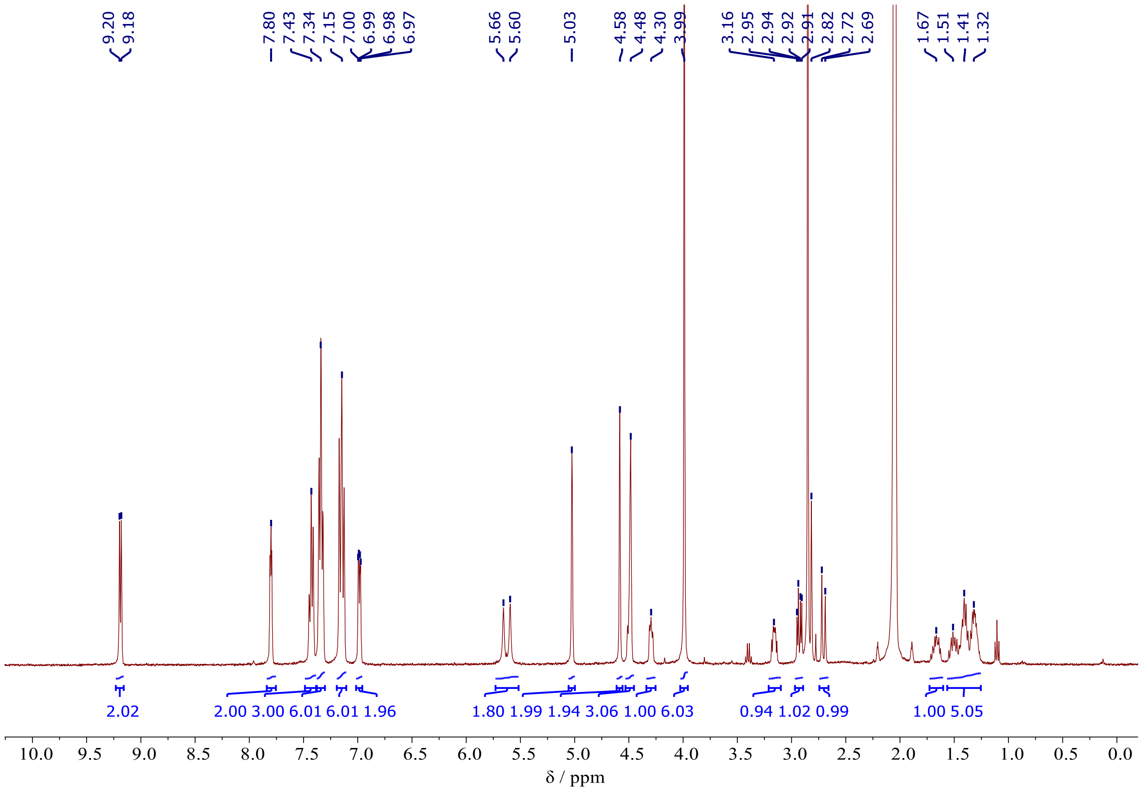

Figure S16. ^{1}H-NMR spectrum of compound **6** in acetone-d_6 at 298 K.

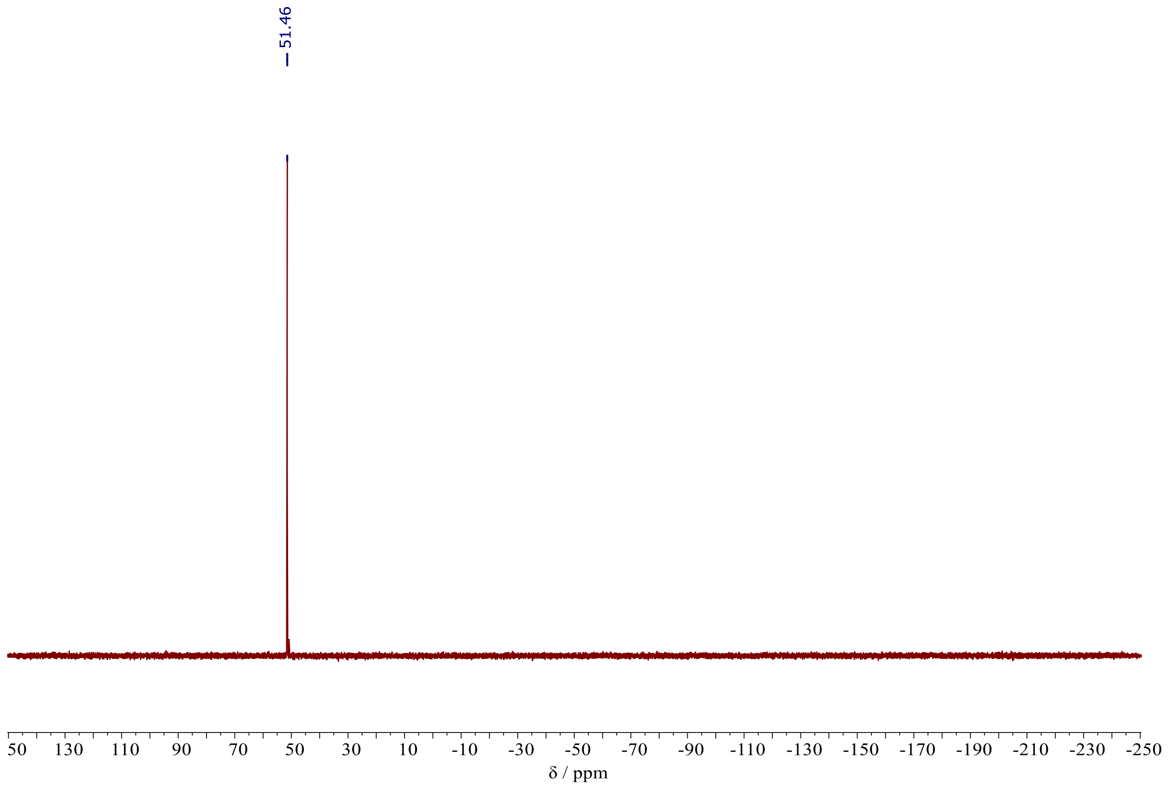

Figure S17. $^{31}P\{^1H\}$-NMR spectrum of compound **6** in acetone-d_6 at 298 K.

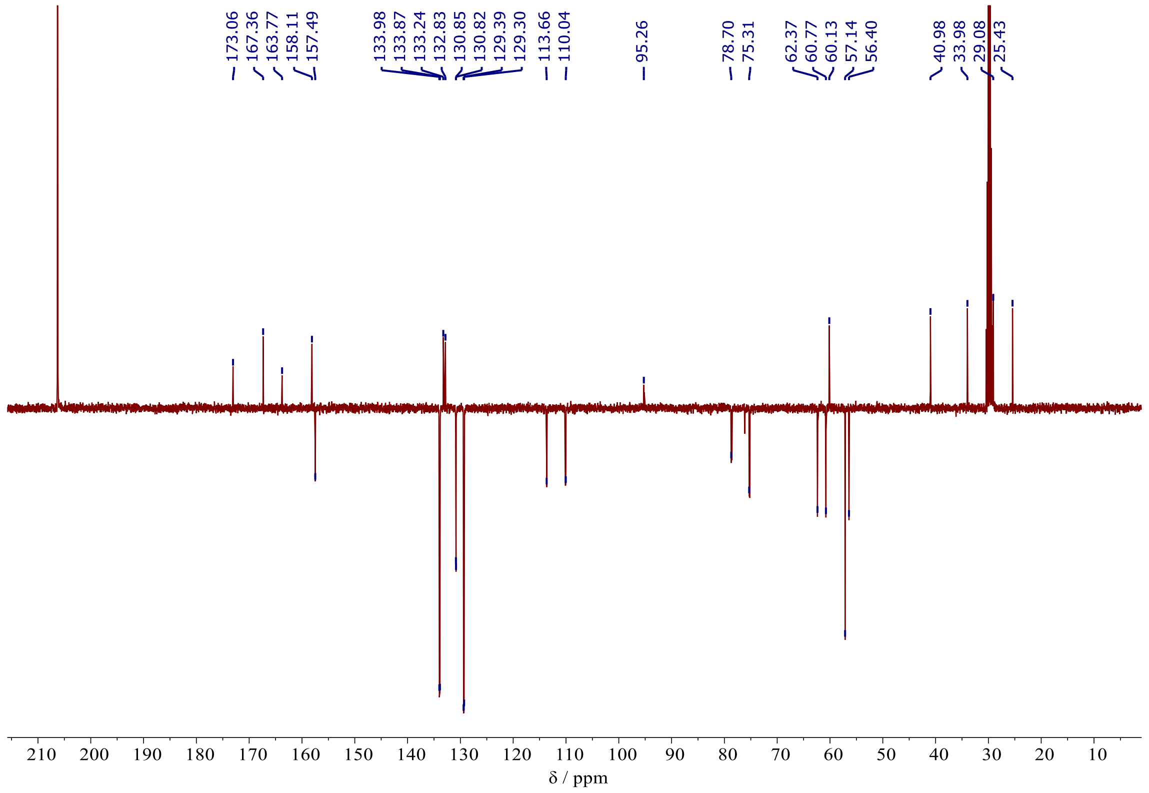

Figure S18. APT $^{13}C\{^1H\}$-NMR spectrum of compound **6** in acetone-d_6 at 298 K.

Electronic data

Table S1. Electronic absorption data for complexes **1**–**6** in dichloromethane and dimethylsulfoxide. Measurements were performed at room temperature using 10^{-4}-10^{-6} M solutions. [sh = shoulder; wavelength values (λ/nm) and correspondent molar absorptivity coefficient (ε $\times 10^{-3}$/$M^{-1}cm^{-1}$)].

	**Dichloromethane**	**Dimethylsulfoxide**
**(1)**	290 (sh), 355 (sh), 373 (3.9), 450 (sh)	-
**(2)**	268 (sh), 292 (sh), 337 (sh), 393 (4.0)	290 (sh), 380 (7.21), 415 (sh)
**(3)**	271 (24.1), 295 (sh), 347 (sh), 427 (3.9), 470 (sh)	290 (21.2), 342 (sh), 416 (3.9), 472 (sh)
**(4)**	290 (18.4), 347 (sh), 419 (3.9), 480 (sh)	294 (19.5), 350 (sh), 414 (3.6), 488 (sh)
**(5)**	242 (sh), 288 (22.1), 341 (sh), 412 (4.3), 473 (sh)	294 (20.3), 356 (sh), 406 (3.6), 478 (sh)
**(6)**	273 (21.3), 345 (sh), 422 (3.7), 472 (sh)	294 (20.5), 350 (sh), 414 (3.2), 480 (sh)

X-ray crystallographic structure determination

[$C_{42}H_{37}ClOP_2Ru$, 0.5 (C_7H_8)] (**1**)

A total of 1814 frames were collected. The integration of the data using a triclinic unit cell yielded a total of 73923 reflections to a maximum θ angle of 27.51° (0.77 Å resolution), of which 8444 were independent (average redundancy 8.755, completeness = 99.7%, Rint = 3.27%, Rsig = 1.69%) and 7766 (91.97%) were greater than 2σ(F2). The final cell constants of a = 9.931(6) Å, b = 11.014(7) Å, c = 19.043(11) Å, α = 80.10(3)°, β = 77.36(2)°, γ = 65.57(3)°, volume = 1843.(2) $Å^3$, are based upon the refinement of the XYZ-centroids of 9210 reflections above 20 σ(I) with 6.612° < 2θ < 54.98°. Data were

corrected for absorption effects using the Multi-Scan method (SADABS). The ratio of minimum to maximum apparent transmission was 0.941. The calculated minimum and maximum transmission coefficients (based on crystal size) are 0.7013 and 0.7456.

The structure was solved and refined using the Bruker SHELXTL Software Package, using the space group P -1, with Z = 2 for the formula unit, $C_{42}H_{37}ClOP_2Ru$, 0.5 (C_7H_8). The final anisotropic full-matrix least-squares refinement on F^2 with 490 variables converged at R1 = 2.29%, for the observed data and wR^2 = 5.52% for all data. The goodness-of-fit was 1.092. The largest peak in the final difference electron density synthesis was 0.763 $e^-/Å^3$ and the largest hole was -0.515 $e^-/Å^3$ with an RMS deviation of 0.057 $e^-/Å^3$. Based on the final model, the calculated density was 1.446 g/cm^3 and F(000), 826 e^-.

[$C_{36}H_{32}N_2O_3PRu$, CF_3O_3S] (2)

A total of 364 frames were collected. The integration of the data using a triclinic unit cell yielded a total of 45014 reflections to a maximum θ angle of 27.61° (0.77 Å resolution), of which 8680 were independent (average redundancy 5.186, completeness = 99.4%, Rint = 5.46%, Rsig = 5.40%) and 6562 (75.60%) were greater than $2\sigma(F^2)$. The final cell constants of a = 10.7494(8) Å, b = 11.5914(8) Å, c = 16.6384(11) Å, α = 73.086(2)°, β = 73.945(2)°, γ = 77.278(2)°, volume = 1884.0(2) $Å^3$, are based upon the refinement of the XYZ-centroids of 9922 reflections above 20 σ(I) with 5.831° < 2θ < 55.13°. Data were corrected for absorption effects using the Multi-Scan method (SADABS). The ratio of minimum to maximum apparent transmission was 0.826. The calculated minimum and maximum transmission coefficients (based on crystal size) are 0.8200 and 0.9720.

Refinement used the space group P -1, with Z = 2 for the formula unit, $C_{36}H_{32}N_2O_3PRu$, CF_3O_3S. The final anisotropic full-matrix least-squares refinement on F^2 with 463 variables converged at R1 = 5.75%, for the observed data and wR^2 = 15.75% for all data. The goodness-of-fit was 1.120. The largest peak in the final difference electron density synthesis was 2.588 $e^-/Å^3$ and the largest hole was -1.227 $e^-/Å^3$ with an RMS deviation of 0.119 $e^-/Å^3$. On the basis of the final model, the calculated density was 1.449 g/cm^3 and F(000), 836 e^-.

[$C_{36}H_{33}N_2O_3PRu$, CF_3O_3S] (3)

A total of 404 frames were collected. The integration of the data using a triclinic unit cell yielded a total of 37811 reflections to a maximum θ angle of 28.31° (0.75 Å resolution), of which 8505 were independent (average redundancy 4.446, completeness = 99.4%, Rint = 8.57%, Rsig = 8.23%) and 6319 (74.30%) were greater than $2\sigma(F^2)$. The final cell constants of a = 11.746(9) Å, b = 11.896(8) Å, c = 13.437(10) Å, α = 79.153(15)°, β = 86.96(2)°, γ = 68.819(18)°, volume = 1719.(2) $Å^3$, are based upon the refinement of the XYZ-centroids of 8904 reflections above 20 σ(I) with 6.164° < 2θ < 56.55°. Data were corrected for absorption effects using the Multi-Scan method (SADABS). The ratio of minimum to maximum apparent transmission was 0.846. The calculated minimum and maximum transmission coefficients (based on crystal size) are 0.6309 and 0.7457.

Refinement used the space group P -1, with Z = 2 for the formula unit, $C_{36}H_{33}N_2O_3PRu$, CF_3O_3S. The final anisotropic full-matrix least-squares refinement on F^2 with 462 variables converged at R1 = 5.22%, for the observed data and wR^2 = 9.17% for all data. The goodness-of-fit was 1.033. The largest peak in the final difference electron density synthesis was 0.735 $e^-/Å^3$ and the largest hole was -0.819 $e^-/Å^3$ with an

RMS deviation of 0.114 $e^-/Å^3$. Based on the final model, the calculated density was 1.589 g/cm^3 and F(000), 838 e^-.

Table S2. Crystallographic data and structural refinement details for compounds **1**, **2**, and **3**.

	**Compound 1**	**Compound 2**	**Compound 3**
Empirical formula	$C_{45.50}H_{41}ClOP_2Ru$	$C_{37}H_{32}F_3N_2O_6PRuS$	$C_{37}H_{33}F_3N_2O_6PRuS$
Formula weight (g mol^{-1})	802.24	821.74	822.75
Temperature (K)	100(2)	100(2)	100(2)
Crystal system	Triclinic	Triclinic	Triclinic
Space group	P -1	P -1	P -1
a (Å) | α (º)	9.931(6) | 80.10(3)	10.7494(8) | 73.086(2)	11.746(9) | 79.153(15)
b (Å) | β (º)	11.014(7) | 77.36(2)	11.5914(8) | 73.945(2)	11.896(8) | 86.96(2)
c (Å) | γ (º)	19.043(11) | 65.57(3)	16.6384(11) | 77.278(2)	13.437(10) | 68.819(18)
Volume (Å^3)	1843.(2)	1884.0(2)	1719.(2)
Z	2	2	2
Calculated density (g cm^{-3})	1.446	1.449	1.589
Absorption coeficient (mm^{-1})	0.621	0.575	0.630
Goodness-of-fit	1.092	1.120	1.033
R_1 [I>2σ(I)]	0.0229	0.0575	0.0522
wR_2 [I>2σ(I)]	0.0521	0.1469	0.0831

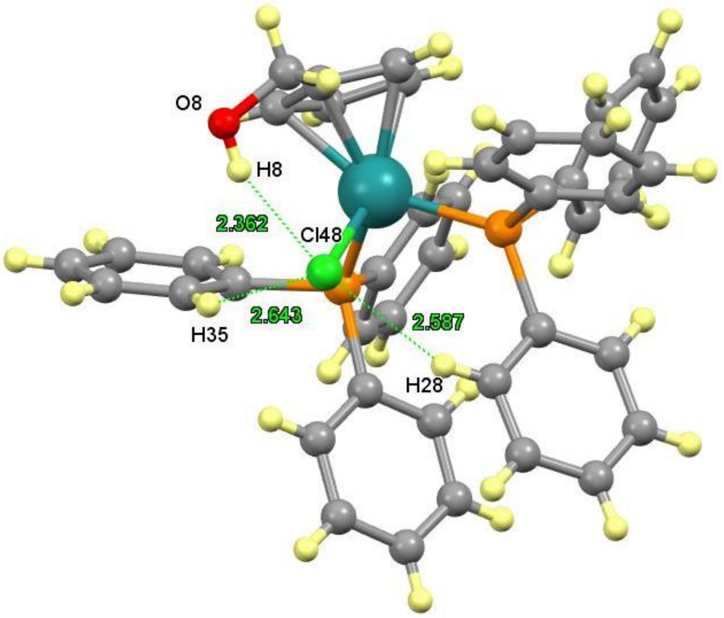

Figure S19. Intramolecular hydrogen bonds observed in compound **1**.

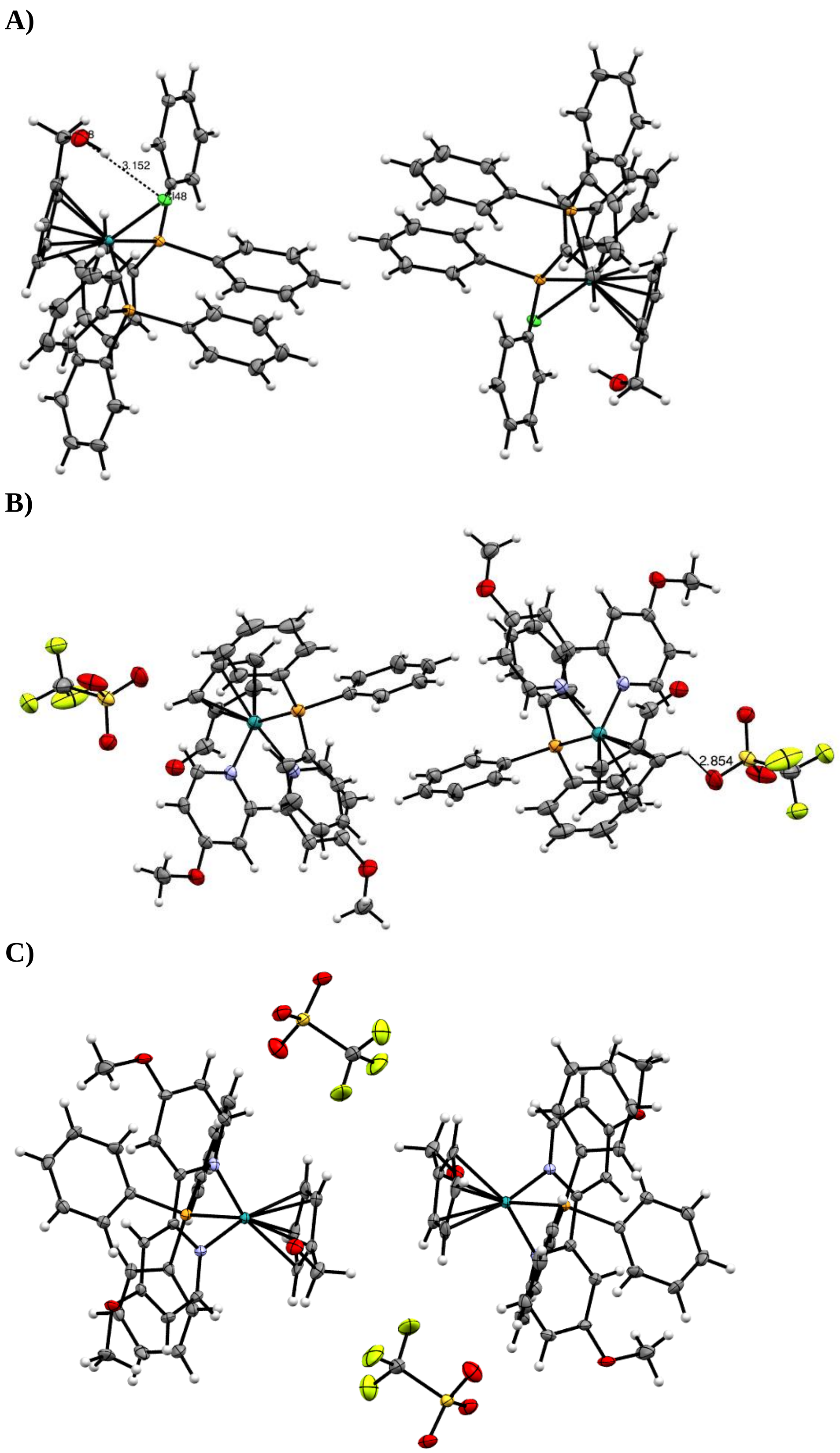

Figure S20. Packing diagram for compounds **1** (**A**), **2** (**B**), and **3** (**C**).

A)

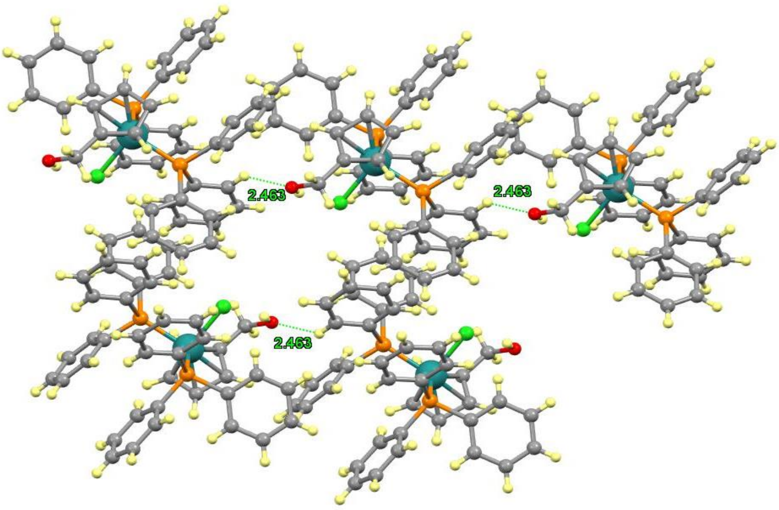

B)

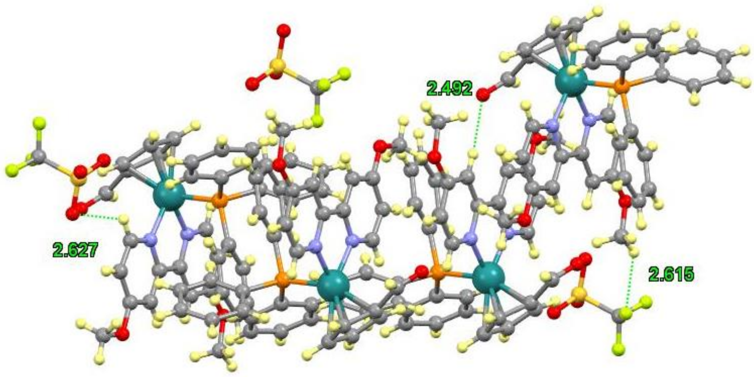

C)

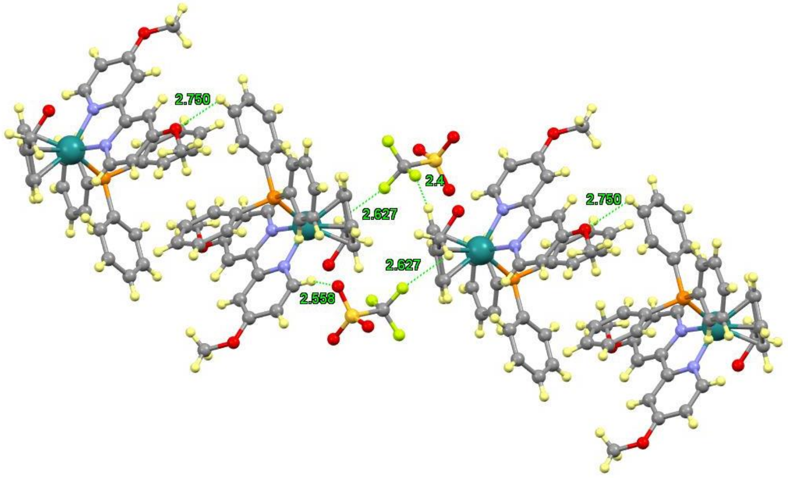

Figure S21. Intermolecular hydrogen bonds in the packing diagrams for compounds **1** (**A**), **2** (**B**), and **3** (**C**).

Stability studies in aqueous solution

A)

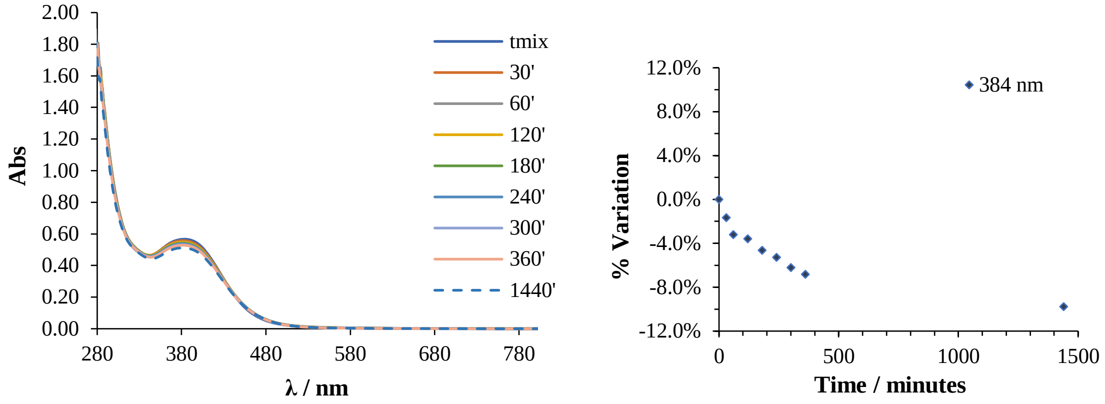

B)

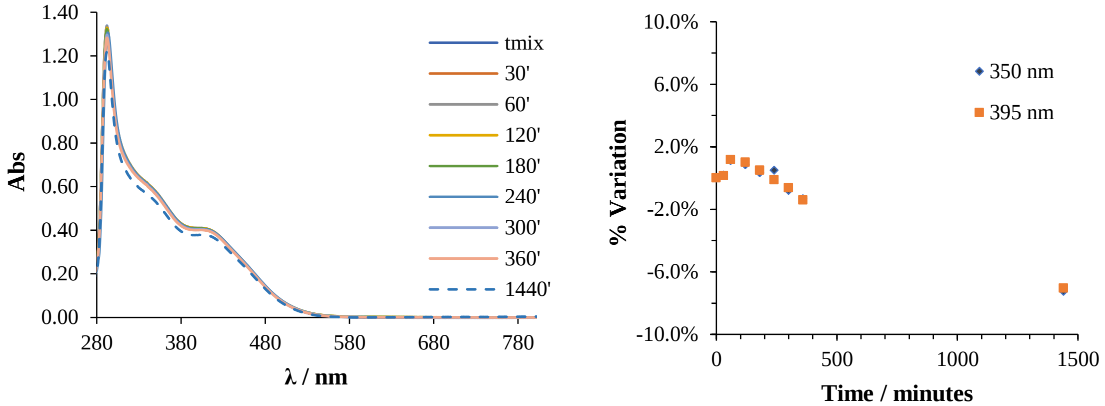

C)

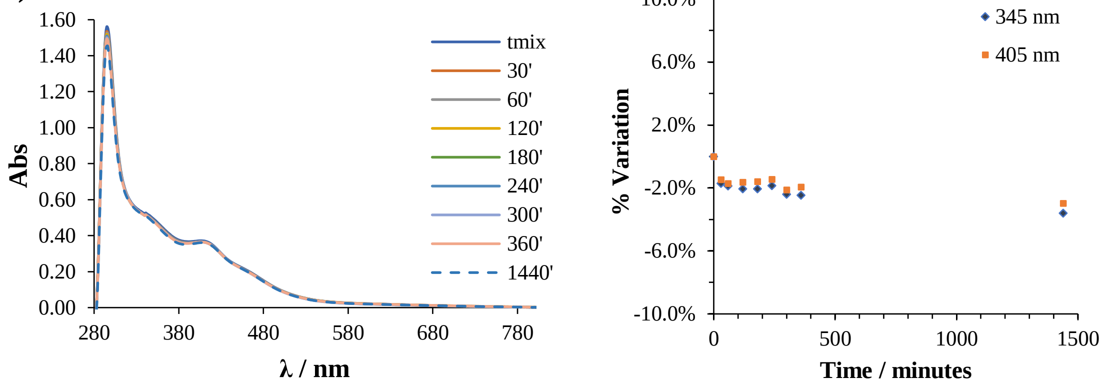

D)

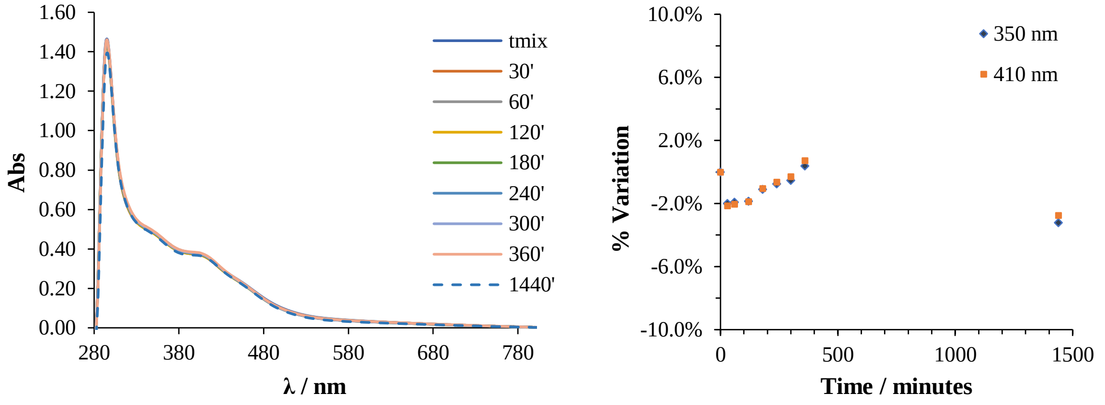

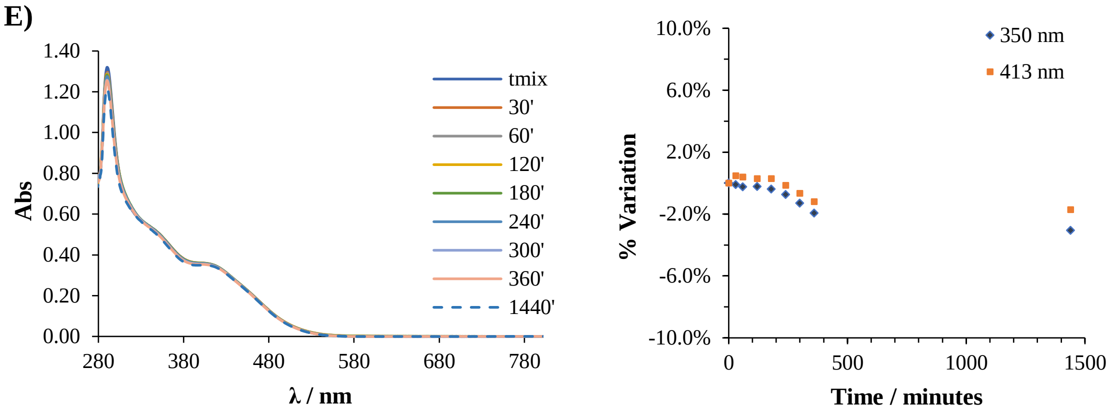

Figure S22. Stability study in cell culture medium (2 % DMSO / 98 % DMEM) for compounds **2** (**A**), **3** (**B**), **4** (**C**), **5** (**D**), and **6** (**E**) at 100–150 µM. On the right: UV–Vis spectra recorded during the 24 h of the study (room temperature); On the left: relative Absorbance Variation (%) at selected wavelengths over time (1 cm optical path; see experimental section for details).

Molecular docking studies

Table S3. List of most prevalent residues involved in the binding sites of all Ru-complexes and P-gp.

R-site	W232, I299, F303, I306, F343, S349, S766, F770, Q773
M-site	F303, I306, Y307, Y310, F343, Q347, Q725, F728, F983, M986, Q990
H-site	H61, Q195, I868, E875, F942, T945, Q946, M949, Q990, S993

Table S4. Molecular Docking binding energies (ΔG) of compound **RT151** on the three binding sites of P-gp where a single residue (Res N) was mutated to alanine. The energy differences to the wild-type P-gp were also computed (ΔΔG). All energies are in kcal/mol.

**R site**			**H site**			**M site**		
wild-type ΔG: -9.3			wild-type ΔG: -7.6			wild-type ΔG: -7.9		
Res N	ΔG (kcal/mol)	ΔΔG	Res N	ΔG (kcal/mol)	ΔΔG	Res N	ΔG (kcal/mol)	ΔΔG
232	-8.2	+1.1	61	-7.6	0.0	303	-7.0	+0.9
299	-8.9	+0.4	195	-7.6	0.0	306	-7.9	0.0
303	-9.7	-0.4	868	-7.3	+0.3	307	-8.0	-0.1
306	-9.7	-0.4	875	-7.7	-0.1	310	-8.3	-0.4
343	-8.9	+0.4	942	-7.5	+0.1	343	-8.7	-0.8
349	-9.2	+0.1	945	-7.7	-0.1	347	-7.0	+0.9
766	-9.8	-0.5	946	-7.5	+0.1	725	-9.0	-1.1
770	-9.9	-0.6	949	-9.2	-1.6	728	-7.3	+0.6
773	-9.3	+0.0	990	-7.3	+0.3	983	-7.5	+0.4
			993	-7.6	+0.0	986	-8.5	-0.6
						990	-7.8	+0.1

Table S5. Molecular Docking binding energies (ΔG) of compound **RT118** on the three binding sites of P-gp where a single residue (Res N) was mutated to alanine. The energy differences to the wild-type P-gp were also computed (ΔΔG). All energies are in *kcal/mol.*

**R site**			**H site**			**M site**		
wild-type ΔG: -8.7			wild-type ΔG: -7.2			wild-type ΔG: -8.1		
Res N	ΔG (kcal/mol)	ΔΔG	Res N	ΔG (kcal/mol)	ΔΔG	Res N	ΔG (kcal/mol)	ΔΔG
232	-7.6	+1.1	61	-7.2	+0.0	303	-7.4	+0.8
299	-8.6	+0.2	195	-7.2	-0.1	306	-7.8	+0.3
303	-8.5	+0.3	868	-7.3	-0.1	307	-8.4	-0.3
306	-9.3	-0.5	875	-7.4	-0.2	310	-7.9	+0.2
343	-8.2	+0.6	942	-7.5	-0.2	343	-8.8	-0.7
349	-8.6	+0.2	945	-7.4	-0.1	347	-8.0	+0.2
766	-8.7	+0.1	946	-7.3	+0.0	725	-7.9	+0.2
770	-8.6	+0.2	949	-7.8	-0.6	728	-7.9	+0.2
773	-8.7	+0.1	990	-7.8	-0.5	983	-7.5	+0.6
			993	-7.3	-0.1	986	-8.4	-0.3
						990	-7.8	+0.3

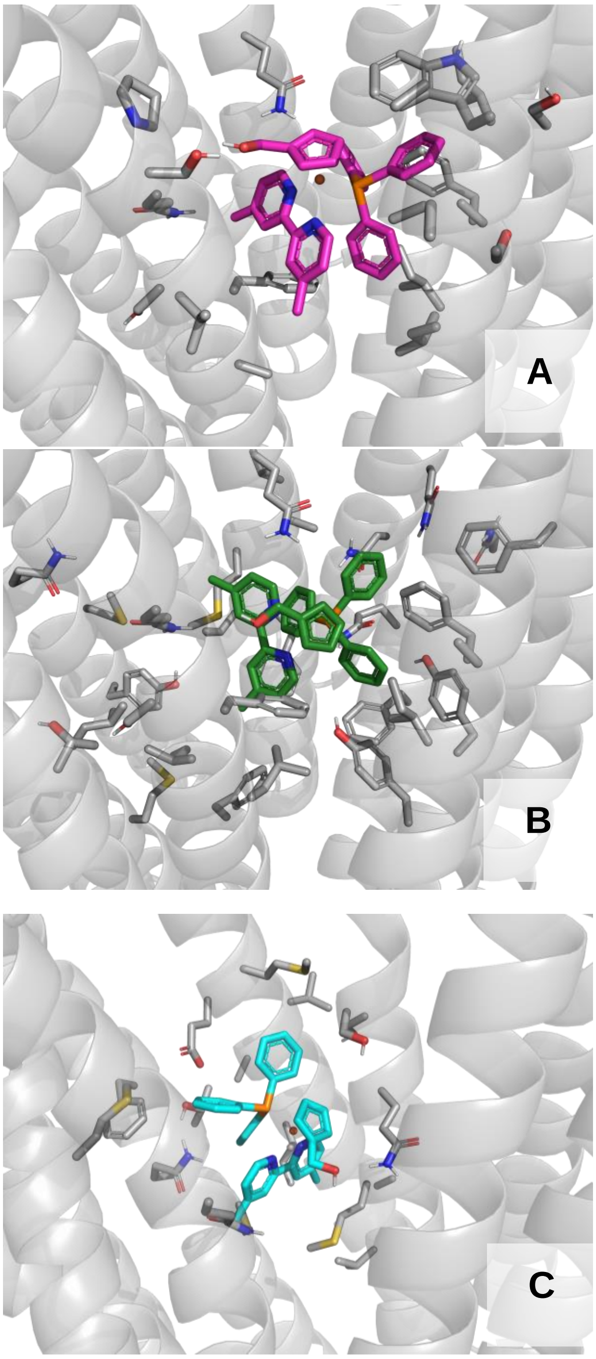

Figure S23. Key P-gp residues within 5 Å of the best **RT151** docking pose on the R (**A**), M (**B**), and H (**C**) binding sites (magenta, green, and cyan **RT151** respectively). P-gp is shown as a grey cartoon and the key residues are shown as sticks.

Table S6. List of residues involved in each of the P-gp binding sites. Residues at the interface can be involved in both of those sites.

M-site	66, 69, 73, 303, 306, 307, 310, 311, 314, 332, 333, 336, 339, 340, 343, 347, 721, 724, 725, 728, 729, 732, 759, 762, 766, 835, 838, 839, 842, 971, 975, 978, 979, 983, 986, 990
R-site	229, 232, 233, 236, 240, 291, 292, 295, 296, 298, 299, 300, 303, 306, 342, 343, 345, 346, 349, 766, 770, 773, 774, 778
H-site	58, 61, 62, 64, 65, 121, 122, 125, 128, 129, 132, 133, 188, 191, 192, 195, 196, 199, 864, 865, 868, 869, 872, 875, 876, 879, 938, 939, 941, 942, 945, 946, 949, 982, 985, 986, 989, 990, 992, 993

HPLC analysis

Reverse phase (RP)-HPLC was performed to assess the purity of complexes **2**, **3**, **RT150** and **RT151**. The complexes were dissolved in methanol and filtered with 20 μm filters. Chromatographic analyses were run on an HPLC Ultimate 3000 Dionex system (Dionex Co., Sunnyvale, CA) using a Luna C18 (2) column (250 mm × 4.6 mm; 5 μm; Phenomenex, Torrance, CA).

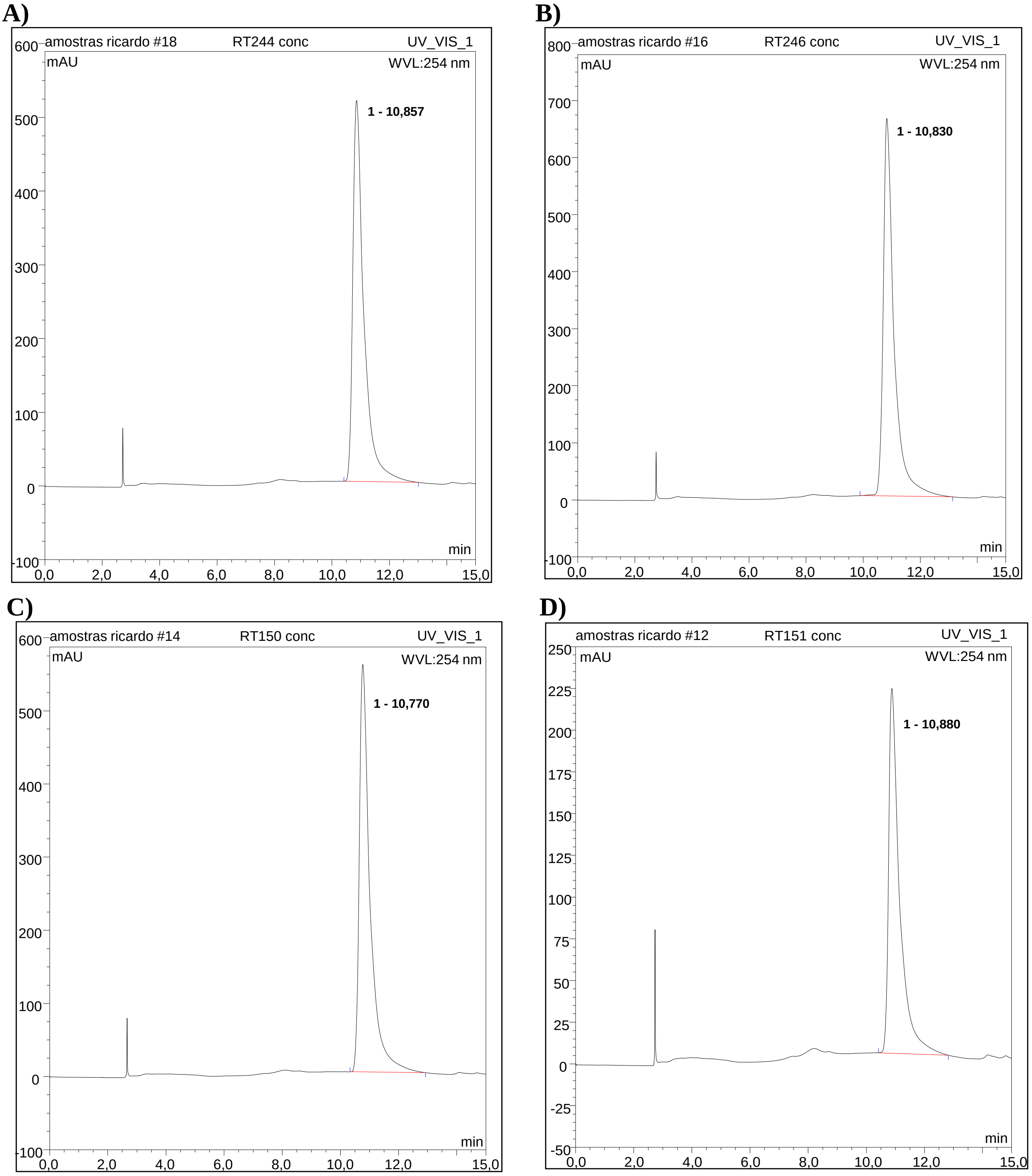

Figure S24. HPLC traces of complexes **2** (**A**), **3** (**B**), **RT150** (**C**) and **RT151** (**D**).

References

[a] Tânia S. Morais, Filipa Santos, Leonor Côrte-Real, Fernanda Marques, M. PaulaRobalo, Paulo J. Amorim Madeira, M. Helena, Garcia, "Biological activity and cellular uptake of [Ru(η^5-C_5H_5)(PPh_3)(Me_2bpy)][CF_3SO_3] complex.", *Journal of Inorganic Biochemistry*, 122 (**2013**), 8-17.
DOI: 10.1016/j.jinorgbio.2013.01.011.

[b] Leonor Côrte-Real, Ricardo G. Teixeira, Patrícia Gírio, Elisabeta Comsa, Alexis Moreno, Rachad Nasr, Hélène Baubichon-Cortay, Fernando Avecilla, Fernanda Marques, M. Paula Robalo, Paulo MendeJoão P. Prates Ramalho, M. Helena Garcia, Pierre Falson**,** Andreia Valente, "Methyl-cyclopentadienyl Ruthenium Compounds with 2,2′-Bipyridine Derivatives Display Strong Anticancer Activity and Multidrug Resistance Potential", *Inorganic Chemistry*, 57, 8 (**2018**), 4629–463957.
DOI: 10.1021/acs.inorgchem.8b00358.

[c] Ricardo G. Teixeira, Dimas C. Belisario, Xavier Fontrodona, Isabel Romero, Ana Isabel Tomaz, M. Helena Garcia, Chiara Riganti, Andreia Valente, "Unprecedented collateral sensitivity for cisplatin-resistant lung cancer cells presented by new ruthenium organometallic compounds", *Inorganic Chemistry Frontiers*, 8 (**2021**) 1983-1996.
DOI: 10.1039/D0QI01344G.
